# Machine learning—based analysis of blood biomarker features in spastic cerebral palsy and their clinical significance

**DOI:** 10.3389/fneur.2026.1797944

**Published:** 2026-03-23

**Authors:** Yanjun Mo, Yu Jiang, Zhaozhan Qiang, Jiashu Yue, Ying Zeng, Lin Xu, Xiaoye Li, Xiaohong Mu

**Affiliations:** 1 Dongzhimen Hospital, Beijing University of Chinese Medicine, Beijing, China; 2 Hubei University of Chinese Medicine, Wuhan, China; 3 Xi'an Children's Hospital, Xi'an, China

**Keywords:** gross motor function classification system, hematological parameters, machine learning, mean platelet volume, spastic cerebral palsy

## Abstract

**Background:**

Cerebral palsy (CP) represents the most prevalent motor disability in childhood, with spastic cerebral palsy (SCP) constituting the predominant subtype. However, systematic characterization of differences in systemic inflammatory status and metabolic profiles between children with SCP and healthy peers remains limited. Here, we applied an interpretable machine-learning framework to evaluate and identify clinically informative inflammation- and metabolism-related biomarkers in children with SCP, thereby providing potential implications for disease monitoring and informing targeted intervention strategies.

**Methods:**

In this retrospective study, 330 children with spastic cerebral palsy (SCP) and 150 healthy controls were enrolled. Complete blood count and serum biochemical parameters were collected, from which 10 systemic immune–inflammation indices were derived. Feature preselection was performed using least absolute shrinkage and selection operator (LASSO) regression, followed by univariable and multivariable logistic regression to identify biomarkers independently associated with the outcome. Model interpretability was assessed using SHapley Additive exPlanations (SHAP), and feature importance was ranked according to SHAP values. Restricted cubic splines (RCS) were applied to evaluate potential nonlinear associations between key indicators and outcome risk, while receiver operating characteristic (ROC) curves were used to assess discriminative performance. Additionally, children with SCP were stratified into severe and mild subgroups according to the Gross Motor Function Classification System (GMFCS) levels, and inflammatory and biochemical differences across severity strata were analyzed. Data were split in a 7:3 ratio using outcome-stratified sampling, with the training set used for model development and the test set for independent performance validation.

**Results:**

Multivariable logistic regression identified 7 independently associated biomarkers: MPV, CHO, DBIL were protective factors, whereas PDW, BASO%, GLB, MCHC were risk factors. A nomogram constructed based on these biomarkers demonstrated favorable performance in discriminating SCP from controls; in the independent test set, the AUC was 0.972 (95% CI, 0.935–0.998). In the SCP subgroup analysis, 330 children were stratified by GMFCS into a severe group (*n* = 160, levels 4–5) and a mild group (*n* = 170, levels 1–3). Multivariable logistic regression indicated that ALT and WBC were positively associated with severe cerebral palsy, whereas ALP showed a weak negative association. The subgroup model yielded an AUC of 0.717 (95% CI, 0.615–0.817) in the independent test set (*n* = 99), indicating modest discriminative ability and thus should be interpreted as exploratory.

**Conclusion:**

This study systematically characterized the inflammation- and metabolism-related profiles that distinguish children with spastic cerebral palsy (SCP) from healthy controls and identified biomarkers associated with disease severity. Indicators such as mean platelet volume (MPV) and platelet distribution width (PDW) may serve as potential biological correlates for monitoring disease status and evaluating intervention responses in SCP.

## 1 Introduction

Cerebral palsy (CP) is defined as a lifelong physical disability affecting movement and posture that results from non-progressive brain injury caused by factors such as prematurity, difficult delivery, asphyxia, or jaundice. Motor impairments in CP are frequently accompanied by disturbances in sensation, perception, cognition, communication, and behavior, and may also coexist with epilepsy and secondary musculoskeletal complications (1). In high-income countries, the prevalence has declined from 2.1 to 1.6 per 1,000 live births, representing a 40% reduction. However, the prevalence remains high in low- and middle-income countries. Spastic cerebral palsy (SCP) accounts for approximately 80% of CP cases (2, 3) and is characterized by increased muscle tone, hyperreflexia, and muscle spasticity, often leading to limb deformities; in severe cases, normal ambulation may be lost. Moreover, with increasing age, metabolic dysregulation and immune imbalance—beyond secondary musculoskeletal problems—gradually become evident, substantially compromising quality of life (4). Nevertheless, current clinical management primarily emphasizes physical rehabilitation, whereas targeted strategies addressing systemic inflammation and metabolic disturbances in SCP remain insufficient, and a comprehensive biomarker landscape has yet to be established.

Inflammation is increasingly recognized as an important contributor to central nervous system (CNS) injury in the developing brain. In addition, spasticity, pain, fatigue, and other secondary manifestations of spastic cerebral palsy may induce metabolic alterations (5, 6). Accordingly, metabolism-related research provides a basis for elucidating the complex pathophysiology of cerebral palsy and for developing strategies to identify potential biomarkers (7). Chronic inflammation and metabolic dysfunction in individuals with cerebral palsy interact bidirectionally and jointly contribute to disease progression. Meanwhile, owing to a combination of factors such as feeding difficulties, malnutrition, and restricted mobility, affected children frequently exhibit abnormalities in complete blood count and biochemical parameters. As the most routine and cost-effective tests available in primary healthcare settings, complete blood count and biochemical assays are widely used in pediatric health care and disease screening (8). These conventional indicators constitute valuable clinical tools for disease monitoring and management; therefore, in-depth investigation of blood-based parameters may yield insights into the pathological basis of cerebral palsy and facilitate the identification of potential targets for intervention.

With the widespread application of machine learning in biomedical research, constructing interpretable predictive models based on multidimensional clinical data has become an important approach to discovering disease biomarkers and analyzing pathological mechanisms. Currently, the SCP clinical management lacks a systematic quantitative assessment tool for systemic inflammation and metabolic abnormalities. Clinicians face difficulty in timely identifying changes in inflammation load, nutritional status, and metabolic disorders in children based on routine test data, which in turn affects the adjustment of intervention plans. This study aims to integrate clinical routine inflammatory markers, biochemical indicators, and composite inflammatory indicators to construct a systemic inflammation-metabolism comprehensive feature map for cerebral palsy patients. Using interpretable machine learning models, the study will screen key independent features that distinguish between cerebral palsy and normal populations, as well as varying degrees of motor function impairment, and reveal their relationship with the disease. The goal is to provide new theoretical foundations and clinical tools for disease monitoring and individualized treatment in SCP.

## 2 Methods

### 2.1 Study population and data collection

This cross-sectional study included 330 children diagnosed with spastic cerebral palsy (SCP) and 158 typically developing (healthy) children. Participants were evaluated between January 2018 and January 2025 at Dongzhimen Hospital, Beijing University of Chinese Medicine, and Xi’an Children’s Hospital.

Inclusion criteria: The diagnosis of SCP was established according to the definition, classification, and diagnostic criteria recommended in 《Cerebral Palsy: Modern Surgical Treatment and Rehabilitation》 (9). Eligible participants were aged 3–12 years.

Exclusion criteria: (1) other subtypes of cerebral palsy; (2) presence of severe medical comorbidities involving the heart, lungs, liver, kidneys, or other major organ systems; (3) cerebral palsy attributable to other etiologies (e.g., infection, jaundice, etc.); (4) other conditions that may cause cerebral palsy–like manifestations, including genetic, metabolic, or neurodegenerative disorders, brain tumors, traumatic brain injury, and related diseases; (5) current use of medications or therapeutic interventions that could affect study outcomes, such as immunosuppressants (e.g., methotrexate, azathioprine), long-term systemic glucocorticoids, anticoagulants (e.g., warfarin, heparin), antiplatelet agents, or recently initiated iron supplementation and other drugs known to influence hematological parameters; (6) hematologic disorders such as immunodeficiency or coagulation abnormalities; and (7) history of acute infection or fever within the preceding 2 weeks.

Inclusion and exclusion criteria for the healthy control group: Inclusion criteria: Children aged 3–12 years with normal neurodevelopment confirmed by clinical evaluation and no abnormalities on physical examination. Exclusion criteria: (1) presence of severe medical comorbidities involving the heart, lungs, liver, kidneys, or other major organ systems; (2) current use of medications or therapeutic interventions that could affect study outcomes (e.g., immunosuppressants, long-term systemic glucocorticoids, anticoagulants, antiplatelet agents, or recently initiated iron supplementation and other drugs known to influence hematological parameters); (3) hematologic disorders such as immunodeficiency or coagulation abnormalities; and (4) history of acute infection or fever within the preceding 2 weeks.

This study was approved by the Ethics Committee of Dongzhimen Hospital, Beijing University of Chinese Medicine (Approval No.: 2024DZMEC-292-03). Written informed consent was obtained from all participants and/or their legal guardians.

Through electronic medical record review, general clinical data were collected, including sex, age, and general medical history. Blood laboratory testing provided hematological and biochemical parameters, including platelet count, neutrophil count, lymphocyte count, monocyte count, total cholesterol, triglycerides, high-density lipoprotein, and low-density lipoprotein. Based on these parameters, we calculated ten surrogate immune–inflammation indices: the neutrophil-to-lymphocyte ratio (NLR), platelet-to-lymphocyte ratio (PLR), systemic immune-inflammation index (SII), systemic inflammation response index (SIRI), red cell distribution width-to-platelet ratio (RPR), mean platelet volume-to-platelet ratio (MPR), neutrophil-to-monocyte ratio (NMR), neutrophil-to-albumin ratio (NAR), monocyte-to-lymphocyte ratio (MLR), and aggregate index of systemic inflammation (AISI).

### 2.2 Statistical analysis

All statistical analyses were performed using R (version 4.5.2). Normality of continuous variables was assessed using the Shapiro–Wilk test. Normally distributed variables are presented as mean ± standard deviation (SD), whereas non-normally distributed variables are summarized as median (interquartile range, IQR). Categorical variables are reported as counts (percentages). Between-group comparisons were conducted using the independent-samples t test or the Mann–Whitney U test for continuous variables, and the χ^2^ test or Fisher’s exact test for categorical variables, as appropriate. After outcome-stratified splitting at a 7:3 ratio, missing values in continuous variables were imputed using the median of the training set, and the same imputation parameters derived from the training set were applied to the test set.

### 2.3 LASSO regression analysis

Feature selection was performed using LASSO-penalized binary logistic regression. In the primary analysis, *λ*.1se (0.008134) was used, whereas λ.min (0.004655) was applied for sensitivity analysis. All laboratory variables and derived indices were standardized. The optimal penalty parameter was determined via 10-fold cross-validation, and features with non-zero coefficients were retained and ranked according to the absolute values of their coefficients.

A total of 488 participants (158 healthy controls and 330 children with spastic cerebral palsy) were included and randomly split into a training set (*n* = 341) and a test set (*n* = 147) using outcome-stratified sampling at a 7:3 ratio. All preprocessing procedures were conducted within the training set: missing values in continuous variables were imputed using the training-set median, and Z-score standardization was performed based on the training-set mean and standard deviation. The test set was processed using the imputation and standardization parameters derived from the training set only.

Using 46 candidate variables as inputs, LASSO logistic regression (glmnet, *α* = 1) was implemented for feature selection, with 10-fold cross-validation (type.measure = “class”) employed to select the penalty parameter. The primary analysis adopted *λ*.1se to derive a more conservative feature set with reduced risk of overfitting, while sensitivity analysis using λ.min was conducted to assess the robustness of the findings.

### 2.4 Univariable and multivariable logistic regression analyses

Using the features selected by LASSO as independent variables and group status as the outcome, univariable logistic regression models were fitted for each candidate predictor. Variables with *p* < 0.05 in univariable analyses were subsequently entered into a multivariable logistic regression model to adjust for potential confounding and to identify independently associated features. Results were visualized using forest plots, and statistical significance was defined as *α* = 0.05.

### 2.5 Restricted cubic spline (RCS) analysis

Key indicators identified from the multivariable logistic regression were further examined using restricted cubic splines (RCS; 4 knots). Specifically, an RCS term was incorporated into separate univariable logistic regression models in the training set, with group status (SCP vs. control) as the outcome. Fitted effects were generated using the Predict function, and ggplot2 was used to visualize the potential nonlinear associations between each indicator and the outcome in terms of log-odds. The resulting plots were assembled into a composite figure in a 3 × 3 layout and saved.

### 2.6 SHAP analysis

A random forest model (ntree = 500) was developed on the training set, and SHAP values were computed for samples in the test set using fastshap (nsim = 160, adjust = TRUE). Model predictions were interpreted using SHAP-based visualizations, including bar plots, beeswarm plots, dependence plots, and waterfall plots.

### 2.7 Nomogram development and validation

A multivariable logistic regression model was constructed based on the selected key indicators, and a risk-prediction nomogram was generated using the nomogram function. Model calibration was assessed using the calibrate function with 400 bootstrap resamples, and calibration curves were plotted. Decision curve analysis (DCA) was performed using the decision_curve function to evaluate clinical utility. In addition, ROC curves were calculated for each individual indicator and for the nomogram, and a combined ROC plot was generated to determine the optimal cutoff value.

### 2.8 Multi-model sensitivity analyses

To evaluate the robustness of the primary findings, we conducted multi-model sensitivity analyses under the same outcome-stratified 7:3 train/test split framework as in the primary analysis. For both the primary classification model (healthy controls vs. SCP) and the subgroup model (severe SCP vs. mild SCP), we constructed and compared a generalized linear model (GLM), random forest (RF), and support vector machine (SVM). For the primary model, all three algorithms used the independent features identified by multivariable logistic regression as input variables; likewise, the subgroup model used the independent features determined by multivariable logistic regression as predictors. The training set was used exclusively for model fitting and hyperparameter tuning, whereas the independent test set was used solely for final performance evaluation. For the GLM, binary logistic regression was implemented using stats:glm. For the RF model, the random Forest package was used: within the training set, a grid search over mtry was performed (mtry = 1 to *p*, where *p* denotes the number of features; ntree = 600), and the optimal mtry was selected based on out-of-bag (OOB) error; subsequently, the model was refit on the training set with ntree = 800. For the SVM model, the e1071 package was applied with an RBF kernel: features were standardized using the training-set mean and standard deviation, and hyperparameters were optimized via 5-fold cross-validation over gamma = 2^-4 to 2^1 and cost = 2^-1 to 2^4. Model performance was evaluated only in the test set. We reported the AUC and its 95% confidence interval estimated using bootstrap resampling (1,200 iterations), and provided sensitivity, specificity, and accuracy at the threshold corresponding to the maximum Youden index. Comparisons among the three algorithms were intended solely as sensitivity analyses; the primary inference was based on the logistic regression–based nomogram.

## 3 Results

### 3.1 Refinement of clinical features using LASSO regression

By applying LASSO regression, which is well recognized for its capability in variable selection and regularization, we effectively refined the baseline dataset (baseline characteristics are presented in Supplementary Table S1). In this study, LASSO with 10-fold cross-validation yielded *λ*.mi*n* = 0.004655 and *λ*.1se = 0.008134 among 46 candidate variables. According to the primary analysis criterion (*λ*.1se), 19 features were retained, whereas 23 features were selected under the sensitivity analysis criterion (*λ*.min).

**Figure 1:**
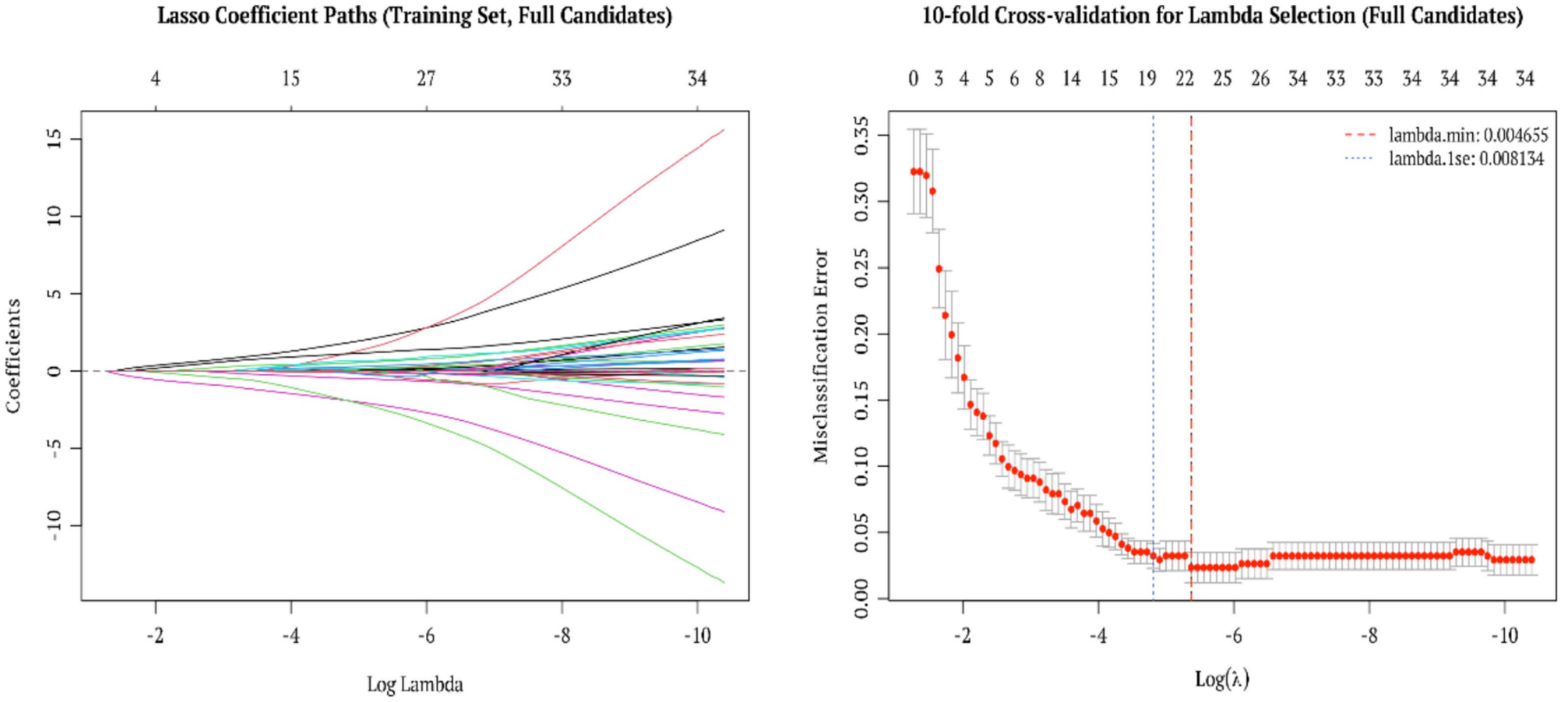
Feature selection results from LASSO regression.

Under λ.1se, the top five features ranked by absolute coefficient magnitude were: MPV (−1.889), DBIL (−1.886), PDW (1.819), TBIL (1.131) and GLB (1.114); under λ.min, the top five were: DBIL (−2.500), PDW (2.246), MPV (−2.227), TBIL (1.753) and GLB (1.243). These results indicate that the ranking of key variables was consistent with prior analytical trends and remained stable under a more stringent training/test separation framework. The corresponding visualizations include the LASSO coefficient path and cross-validation curve (Figure 1), while the coefficient distribution under *λ*.min is provided in Supplementary Figure S1.

### 3.2 Univariable and multivariable analyses of factors associated with cerebral palsy

**Figure 2:**
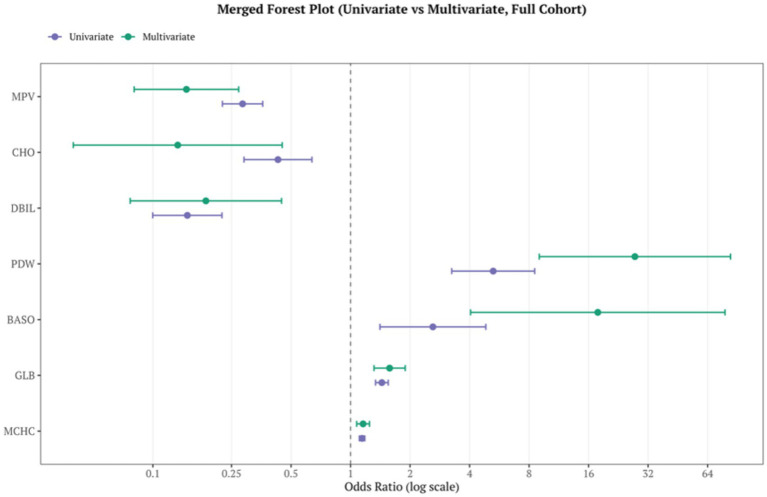
Univariable and multivariable logistic regression analyses of biomarkers independently associated with spastic cerebral palsy.

The results of the univariable and multivariable logistic regression analyses are presented in Figure 2 and Supplementary Figures S2, S3. For the final set of seven core indicators, univariable analyses showed that MPV (OR = 0.284, 95%CI 0.225–0.360), CHO (0.429, 0.289–0.638), DBIL (0.149, 0.100–0.224) were associated with a protective direction, whereas PDW (5.267, 3.248–8.540), BASO% (2.609, 1.408–4.835), GLB (1.440, 1.337–1.551) and MCHC (1.144, 1.108–1.180) were associated with increased risk (all *p* < 0.01,Figure 2).

In the multivariable logistic regression model (full cohort), all seven features remained statistically significant: MPV (OR = 0.148, 95%CI 0.080–0.272), CHO (OR = 0.134, 95%CI 0.040–0.451), DBIL(OR = 0.185, 95%CI 0.077–0.448) were identified as a protective factor, while PDW (OR = 27.443, 95%CI 9.008–83.609), BASO% (OR = 17.816, 95%CI 4.049–78.392), GLB(OR = 1.576, 95%CI 1.314–1.890), MCHC (OR = 1.157, 95%CI 1.074–1.246) were identified as a risk factor (all *p* < 0.01, Figure 2).

### 3.3 Associations between immune–inflammation indicators and spastic cerebral palsy explored using RCS

**Figure 3:**
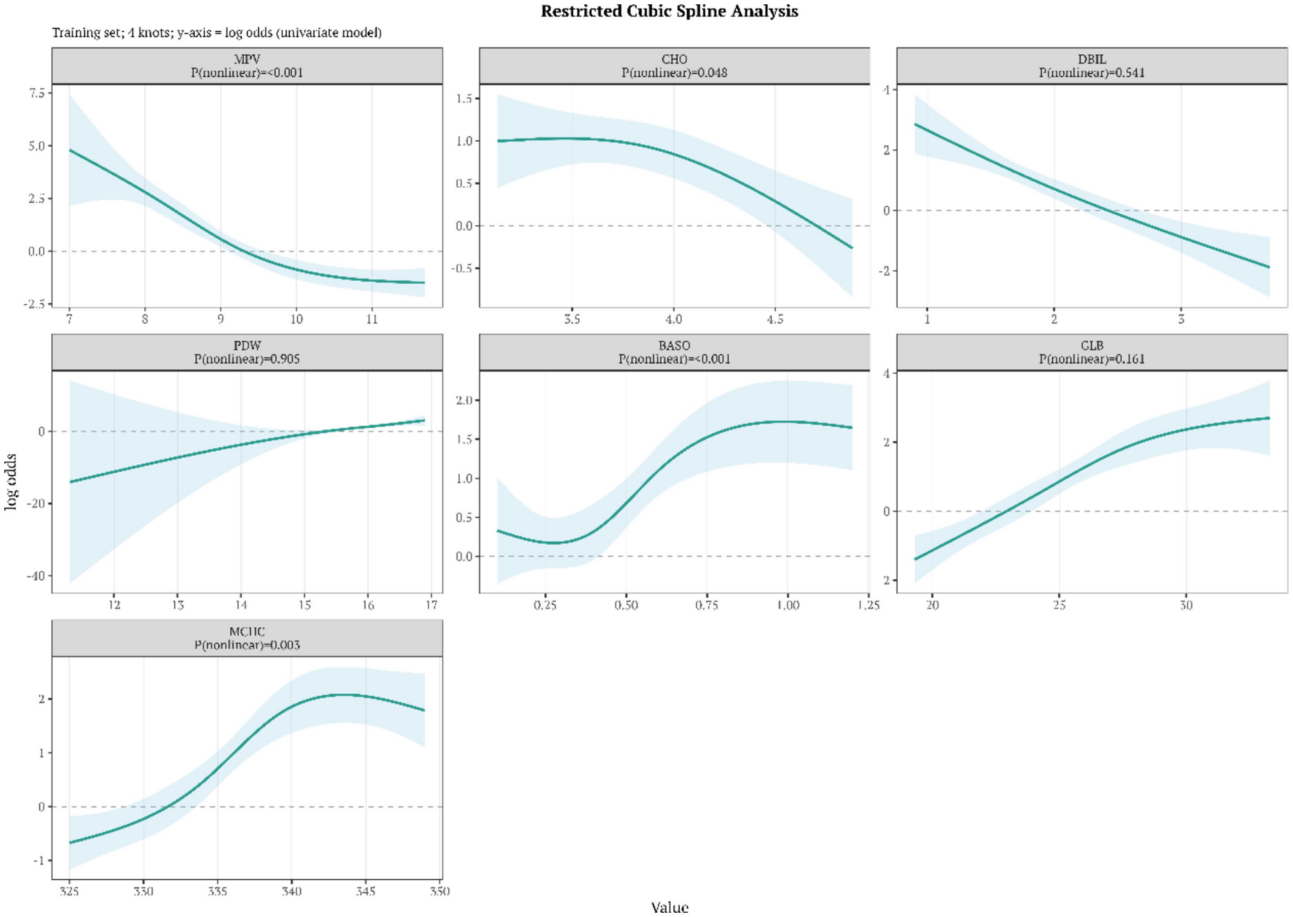
Restricted cubic spline analyses of the associations between seven core indicators and spastic cerebral palsy.

The restricted cubic spline (RCS) analyses indicated that nonlinear associations were not observed for all indicators: MPV (P_nonlinear <0.001), CHO (P_nonlinear = 0.048), BASO% (P_nonlinear <0.001) and MCHC (P_nonlinear = 0.003) exhibited a statistically significant nonlinear relationship, whereas DBIL (0.541), PDW (0.905) and GLB (0.161) showed no significant nonlinearity and was characterized primarily by a linear trend. Specifically, MPV demonstrated an apparent inflection point around 8–9 fL, below which the risk of cerebral palsy increased sharply. BASO% displayed an S-shaped association with an approximate turning point near 0.5%. In addition, the risk associated with MCHC tended to plateau above approximately 330–335 g/L. These thresholds may provide clinically relevant reference values for risk stratification based on routine hematological parameters (Figure 3).

### 3.4 SHAP-based interpretation of feature importance

SHAP values were computed for each feature based on the random forest model, and feature importance was ranked according to the mean absolute SHAP value (Mean |SHAP|) as follows: MPV (0.1170) > DBIL (0.0963) > PDW (0.0904) > GLB (0.0642) > CHO (0.0607) > MCHC(0.0433) > BASO% (0.0150). Among these features, MPV showed the greatest contribution, suggesting a central role in discriminating SCP from healthy controls.

**Figure 4:**
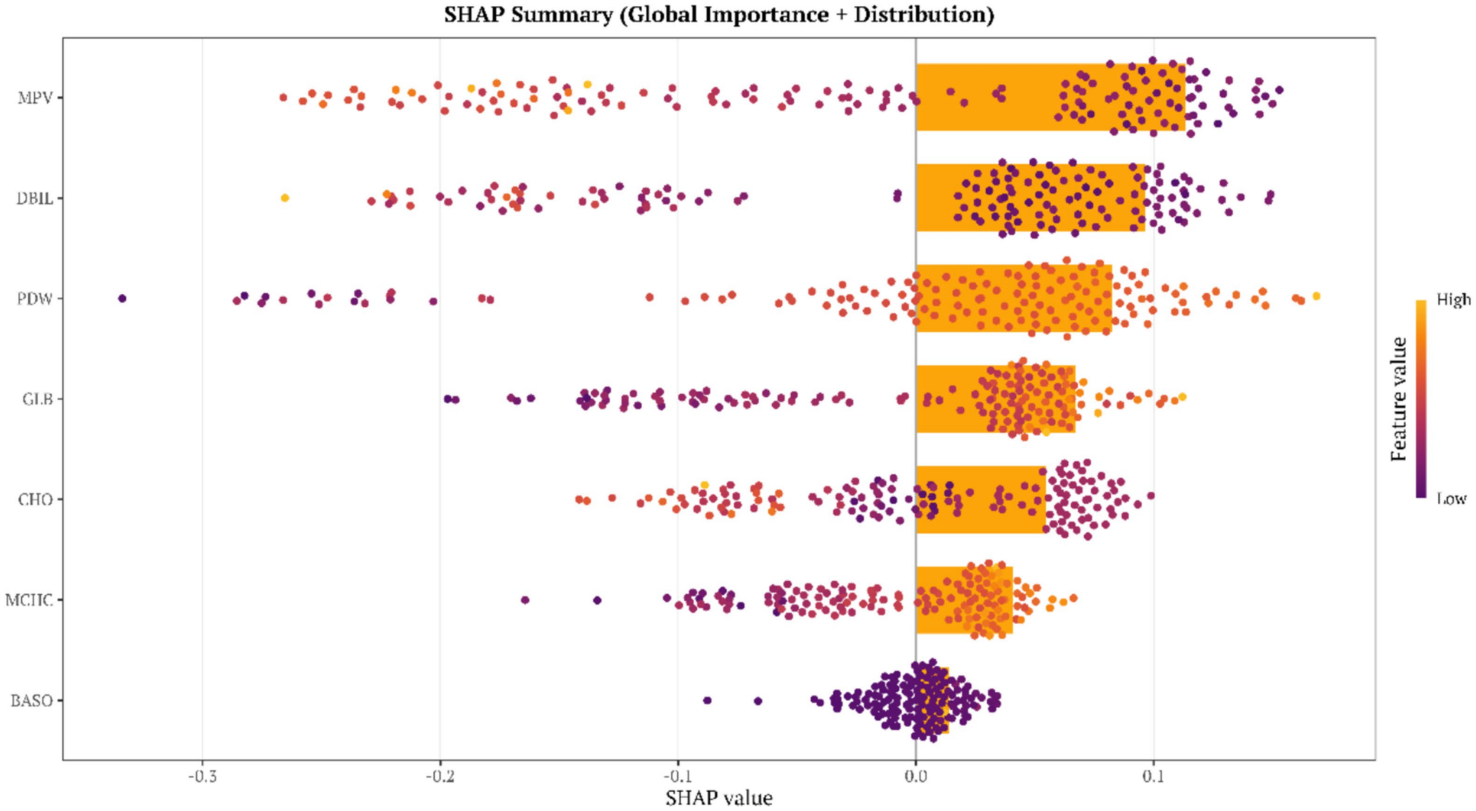
Global SHAP feature importance and SHAP value distribution in the test set.

**Figure 5:**
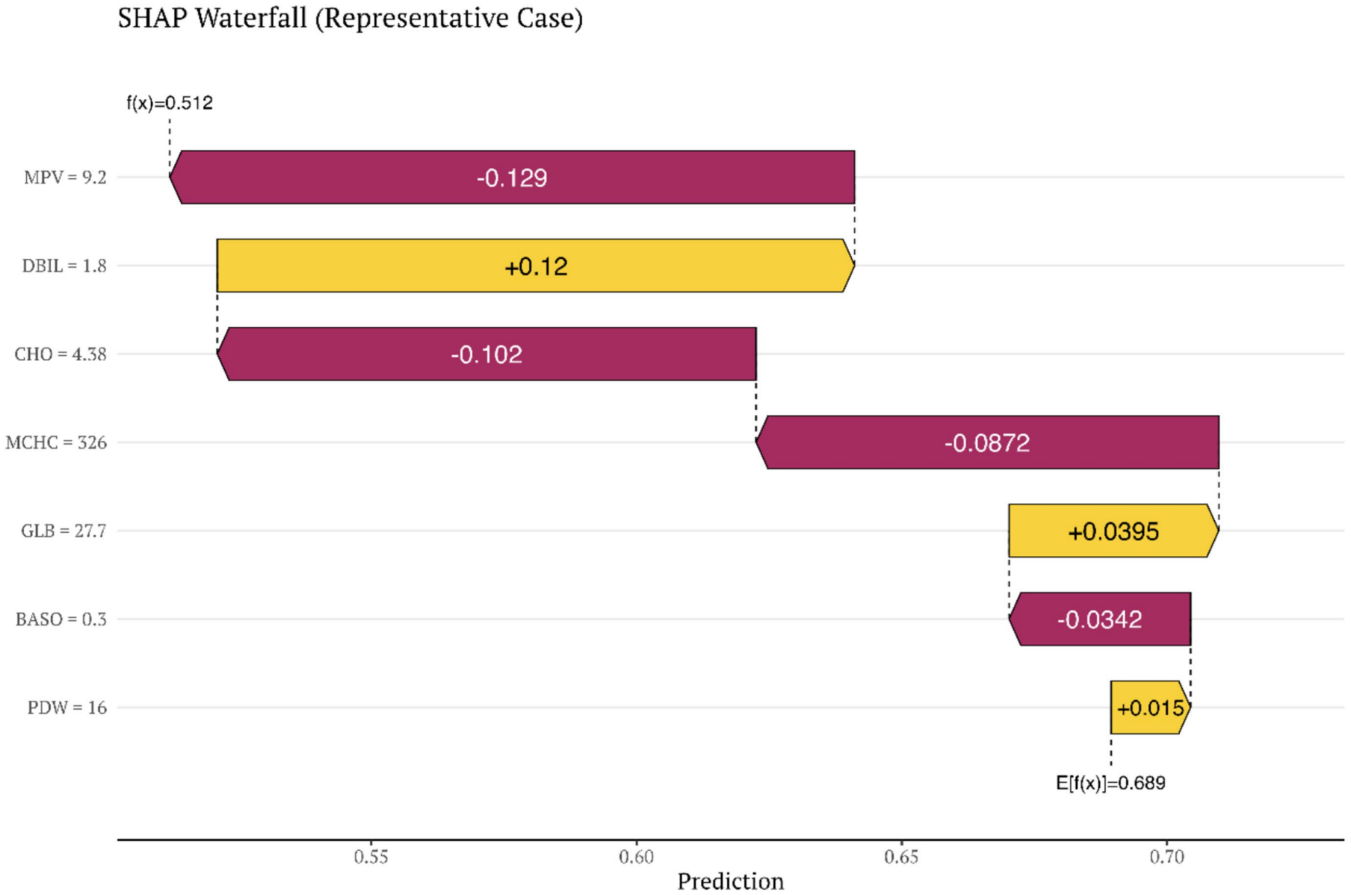
SHAP waterfall plot for a representative individual case with spastic cerebral palsy.

With respect to the directionality of contribution, higher values of PDW, GLB, MCHC, and BASO% contributed positively to disease risk, whereas higher values of MPV, DBIL, and CHO contributed negatively. This pattern was fully consistent with the protective/risk directions identified in the multivariable regression analyses (Figures 4, 5; Supplementary Figures S4–S6).

### 3.5 Nomogram development and validation

**Figure 6:**
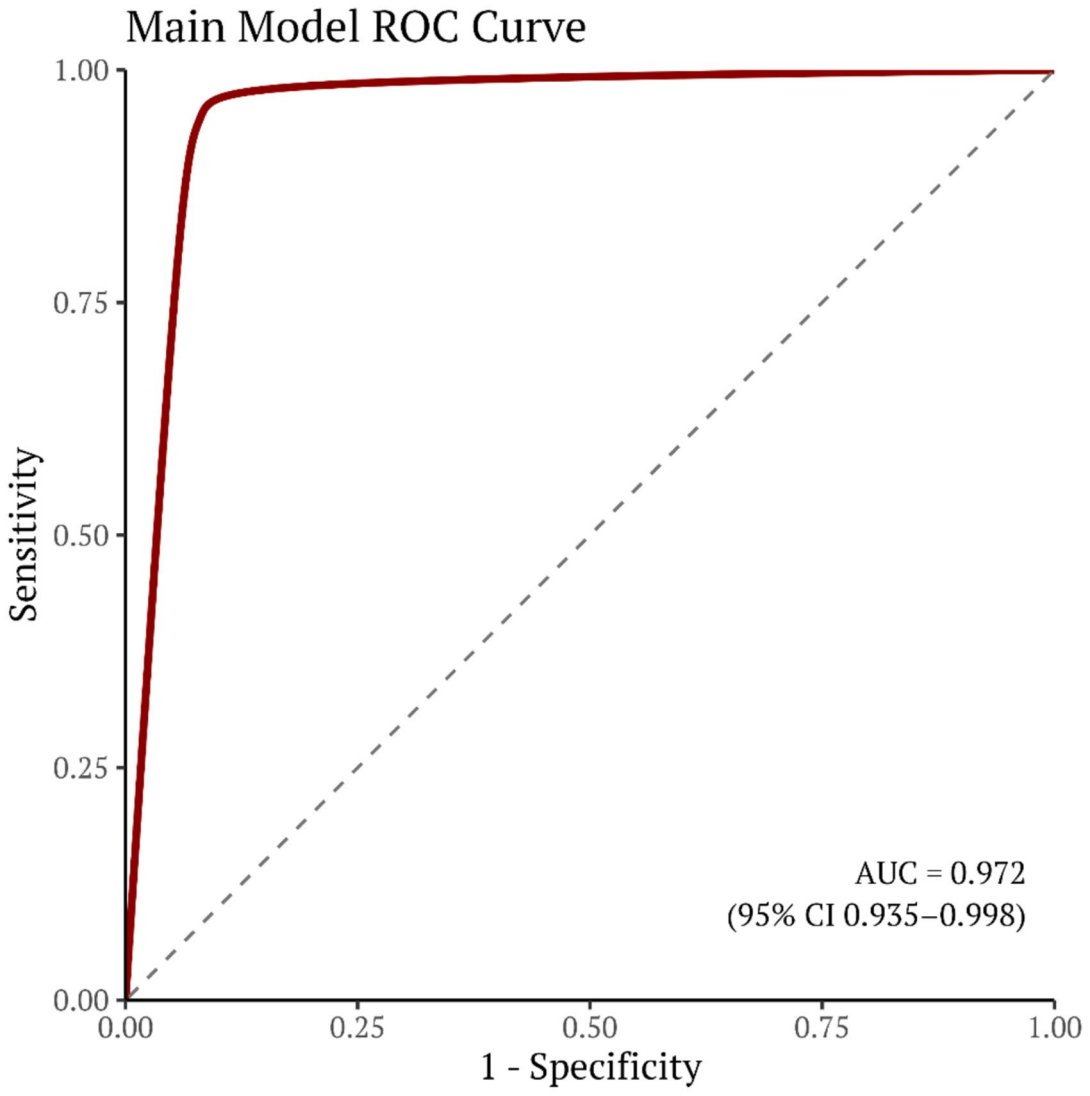
Receiver operating characteristic (ROC) curves of the model.

**Figure 7:**
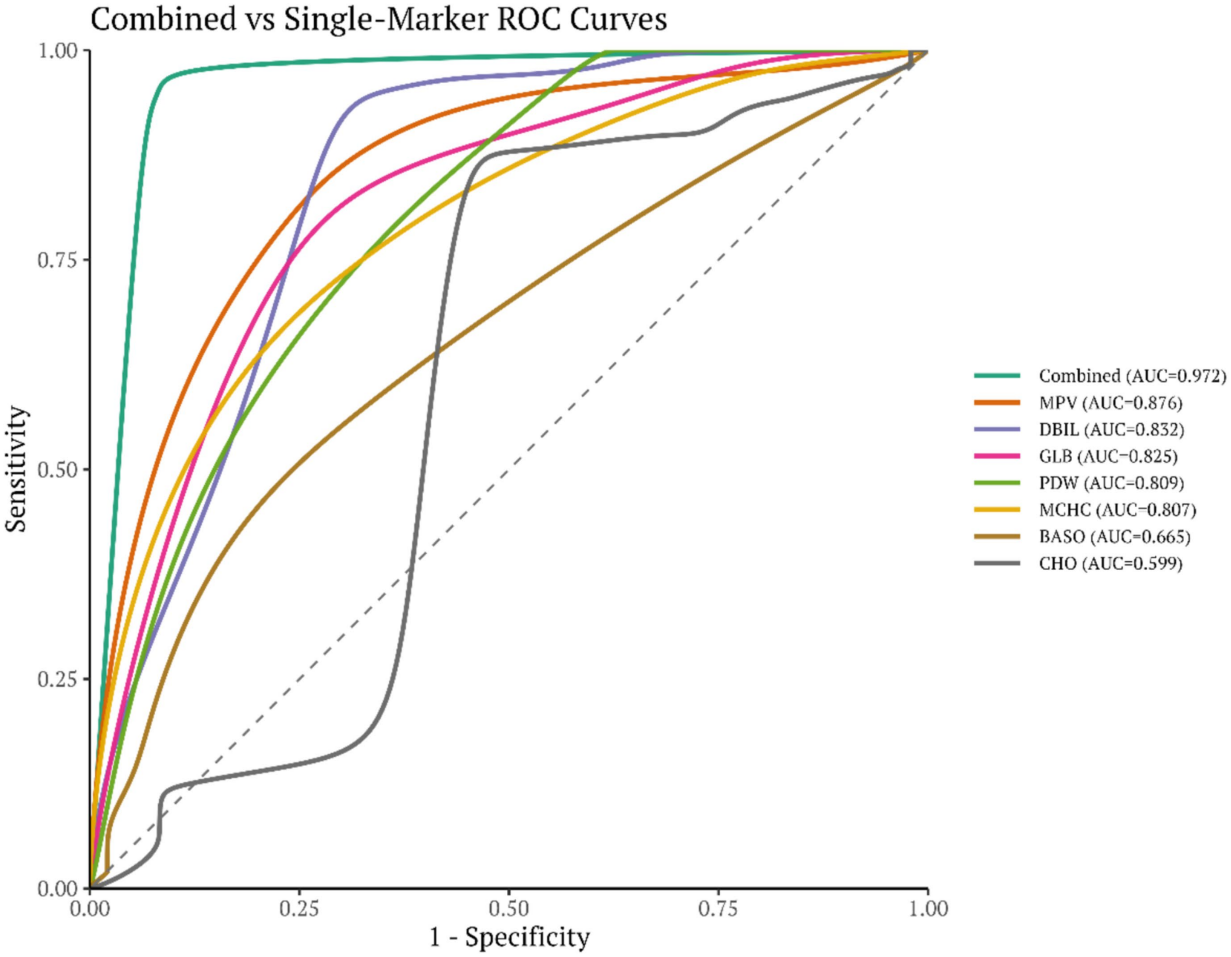
Combined ROC curves for the multi-indicator model and individual indicators.

**Figure 8:**
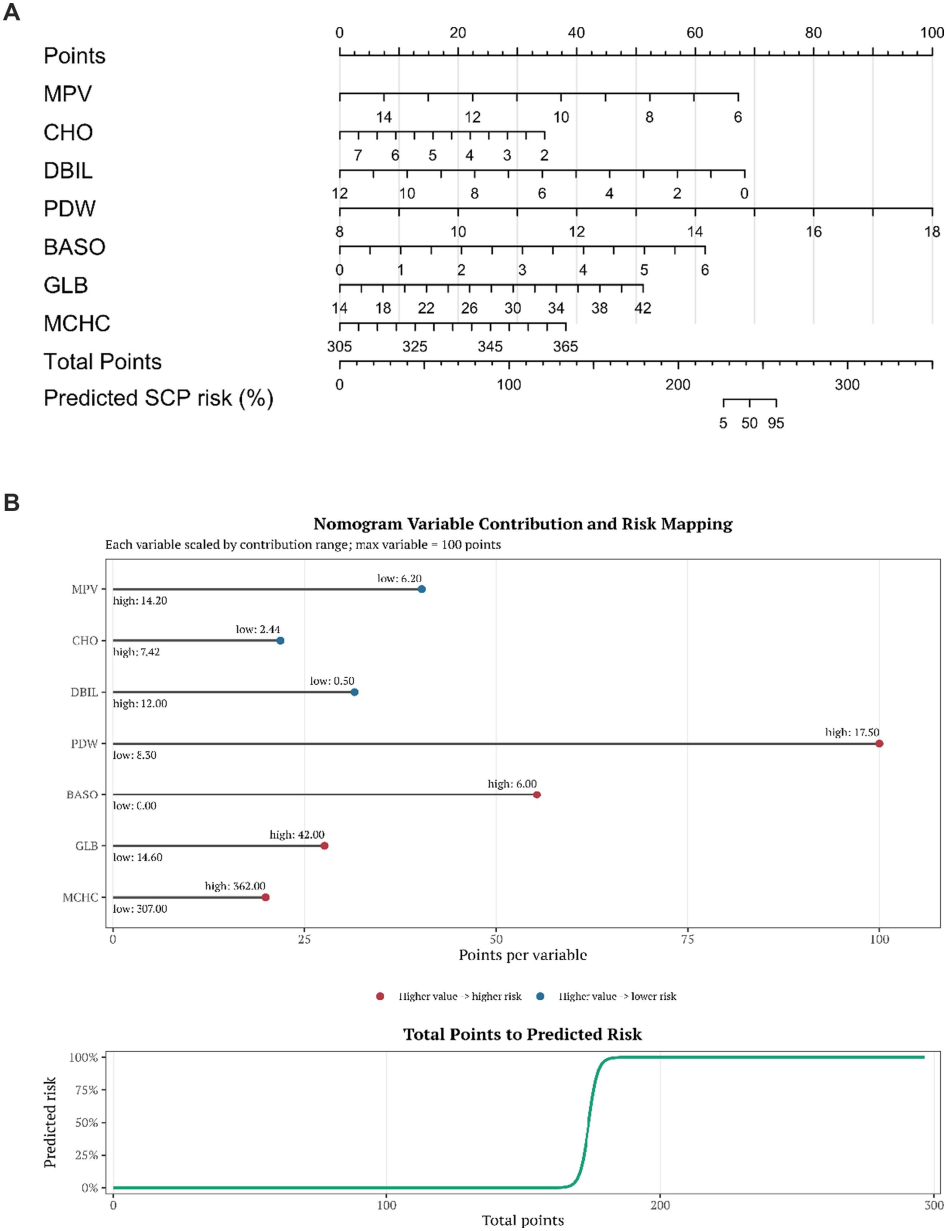
**(A)** Nomogram of the model constructed using seven independent features. **(B)** Variable contributions and risk mapping in the nomogram.

**Figure 9:**
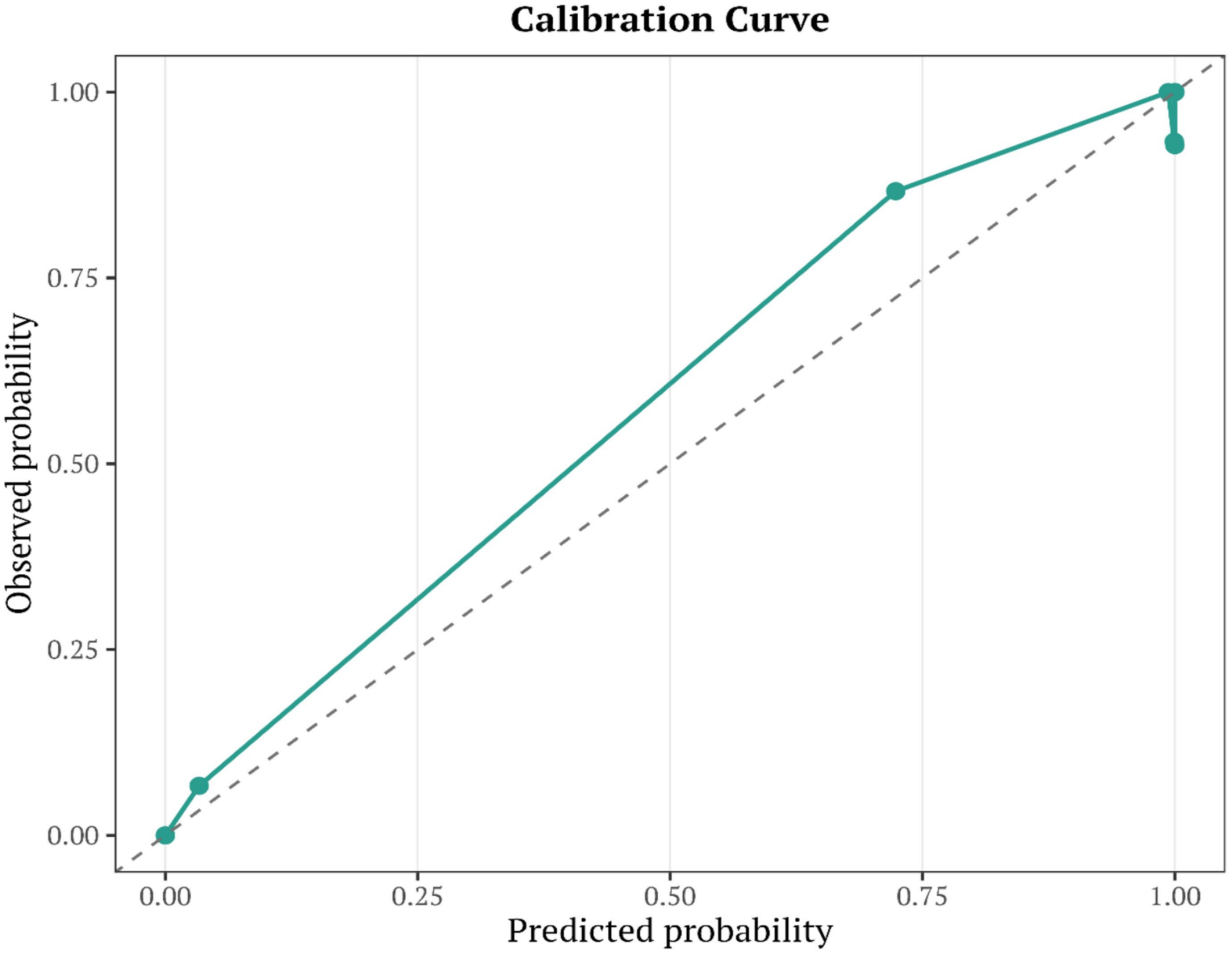
Calibration curve of the model.

A disease risk–prediction nomogram incorporating seven features was successfully developed, enabling rapid estimation of the probability of disease occurrence based on individual indicator values. The calibration curve demonstrated good agreement between predicted and observed probabilities. Decision curve analysis (DCA) further supported the clinical utility of the model across a range of threshold probabilities. In the independent test set (*n* = 147), the model achieved an AUC of 0.972 (95%CI 0.935–0.998, Figure 6). Using the maximum Youden index to determine the optimal cutoff (0.159), the model yielded a sensitivity of 0.990, specificity of 0.917, and accuracy of 0.966. The combined ROC curve indicated that the nomogram model outperformed each individual indicator in terms of AUC, suggesting superior discrimination with multi-indicator integration. The combined ROC curve is shown in Figure 7. The nomogram is presented in Figures 8A,B, and the calibration curve in Figure 9. The DCA plot is provided in Supplementary Figure S7, while the distributions of predicted probabilities and the confusion matrix are shown in Supplementary Figures S8, S9, respectively.

### 3.6 Subgroup analysis

According to GMFCS levels, the 330 children with spastic cerebral palsy were stratified into two groups: a severe group (GMFCS levels 4–5, *n* = 160) and a mild group (GMFCS levels 1–3, *n* = 170). Using the SCP subgroup dataset, univariable and multivariable logistic regression analyses were performed, and an exploratory subgroup nomogram and ROC curves were constructed for preliminary evaluation. Multivariable logistic regression identified three features that remained statistically significant (*p* < 0.05). In the subgroup model (SEVERE vs. MILD), multivariable logistic regression showed that ALT (OR = 1.032, 95% CI 1.008–1.056, *p* = 0.009) and WBC (OR = 1.144, 95%CI 1.037–1.262, *p* = 0.007) were positively associated with severe cerebral palsy, whereas ALP (OR = 0.996, 95% CI 0.992–1.000, *p* = 0.035) exhibited a weak negative association. In the independent test set (*n* = 99), the subgroup model achieved an AUC of 0.717 (95% CI, 0.615–0.817). At the Youden-optimal threshold, the sensitivity, specificity, and accuracy were 0.667, 0.804, and 0.737, respectively, indicating moderate discriminative performance. This result should be interpreted as exploratory and is not intended to serve as an independent clinical decision-making tool. Subgroup analysis results are provided in Supplementary Figures S10–S16.

### 3.7 Multi-model sensitivity analyses

Under the same outcome-stratified 7:3 train/test split framework as the primary analysis, we compared three algorithms: GLM, RF, and SVM. In the primary task (CONTROL vs. SCP), the test-set AUCs were 0.972 for GLM, 0.991 for RF, and 0.971 for SVM. In the subgroup task (severe SCP vs. mild SCP), the test-set AUCs were 0.717 for GLM, 0.552 for RF, and 0.543 for SVM. These results indicate overall concordant discriminative performance across the three models for the primary classification task, whereas GLM outperformed RF and SVM in the subgroup classification task (Supplementary Figures S15–S18). Collectively, these findings support the continued use of the more interpretable logistic regression–based nomogram as the primary analytic model, with the multi-model comparison serving only as a sensitivity analysis. The sensitivity analyses did not alter the main conclusions.

## 4 Discussion

This study, using an interpretable machine-learning framework, identified seven core biomarkers strongly associated with SCP: MPV, CHO, and DBIL as protective factors (significantly decreased in patients with SCP), and PDW, BASO%, GLB, and MCHC as risk factors (significantly increased in patients with SCP). Among them, MPV showed the highest SHAP contribution, highlighting its potentially central pathophysiological role. Restricted cubic spline (RCS) analysis further demonstrated significant nonlinear associations between MPV, BASO%, MCHC, and CHO and SCP, with clinically meaningful inflection points or threshold ranges. In contrast, DBIL, PDW, and GLB exhibited linear associations, suggesting steadily increasing or decreasing effects across the observed value ranges; therefore, sustained deviations in these markers warrant clinical attention. In addition, subgroup analyses indicated positive associations of ALT and WBC with severe grading, whereas ALP showed a weak negative association, suggesting that disease progression may be accompanied by increased burden on specific organ systems. Collectively, these findings reveal multidimensional and interrelated pathophysiological disturbances in children with SCP, providing new targets and ideas for clinical monitoring and intervention.

The seven SCP-associated features identified in this study span multiple pathophysiological dimensions—including coagulation/inflammation, immunity, metabolism, and nutritional status—and together constitute a systemic biomarker panel for spastic cerebral palsy. With respect to coagulation and inflammation, alterations in platelet-related parameters warrant particular attention. Decreased mean platelet volume (MPV) is typically associated with reduced platelet production or diminished platelet reactivity and may reflect impaired megakaryopoiesis or platelet release under chronic inflammatory conditions. RCS analysis showed a significant nonlinear association between MPV and SCP, with an inflection point at approximately 8–9 fL. Below this value, the risk increased sharply, whereas above it the risk tended to plateau, suggesting that this inflection point may serve as a potential clinically relevant cutoff for evaluating platelet function in children with SCP. When a sustained decrease in MPV approaches the threshold during follow-up, clinicians should be alert to the possibility of aggravated chronic inflammation or suppressed bone marrow hematopoietic function. Anti-inflammatory evaluation should be initiated, and factors related to bone marrow suppression (such as the bone marrow toxicity of certain anticonvulsant drugs) should be investigated. In contrast, elevated platelet distribution width (PDW) indicates increased heterogeneity in platelet volume within the peripheral circulation; this is commonly regarded as a marker of enhanced platelet activation and accelerated turnover and is closely linked to hypercoagulability and microvascular endothelial dysfunction (10). Our findings suggest that children with SCP may exhibit an underlying disturbance in the bone marrow hematopoietic microenvironment or chronic inflammation–mediated alterations in platelet kinetics. Increased platelet distribution width, as one of the markers of a hypercoagulable state, combined with the common limitation of limb movement in children, suggests that clinicians should be cautious of the potential for thrombotic diseases and long-term vascular risks. It is recommended to regularly screen for deep vein thrombosis and initiate physical anti-thrombotic interventions when necessary (11). In addition, abnormalities in platelet indices themselves reflect low-grade chronic inflammation. Inflammatory cytokines can disrupt normal megakaryocyte differentiation, leading to increased variability in platelet size, which is consistent with the elevated PDW observed in this study (12).

At the level of immune activation, elevated GLB directly reflects sustained activation of the humoral immune system. GLB represents the aggregate of multiple proteins, including acute-phase proteins, complement components, and immunoglobulins; thus, its increase constitutes a clear signal of chronic systemic inflammation (13). An increased BASO% in children with cerebral palsy further supports the presence of immune dysregulation. As effector cells, basophils participate in allergic responses and anti-parasitic immunity, and fluctuations in their abundance may also be linked to activation of specific chronic inflammatory pathways. RCS analysis showed an S-shaped nonlinear association between BASO% and SCP, with an inflection point at approximately 0.5%. Beyond this level, the risk tended to plateau, consistent with a “dose-to-saturation” immune-effect mechanism, suggesting an upper functional limit to basophil-mediated immune activation. Therefore, regular monitoring of routine blood tests—particularly platelet indices and leukocyte differentials—may help clinicians dynamically evaluate systemic inflammatory burden and immune status, thereby providing a rationale for the timely initiation or adjustment of systemic anti-inflammatory therapy. Notably, some children with comorbid epilepsy require long-term use of antiseizure medications (14), and certain agents (e.g., valproate and carbamazepine) are known to exert complex effects on the immune system and bone marrow hematopoiesis. This may represent a potential confounding factor contributing to subtle changes in blood cell parameters and should be carefully considered when interpreting results in clinical practice (15–17).

Across the metabolic and nutritional dimensions, abnormalities in multiple indices indicate a deeper disruption of homeostasis. Elevated MCHC typically suggests increased hemoglobin concentration within erythrocytes, which may be related to a relative reduction in plasma volume or increased blood viscosity. The latter can increase microcirculatory resistance and compromise tissue perfusion, potentially exacerbating pre-existing ischemic–hypoxic injury in the brain (18). RCS analysis showed a significant nonlinear association between MCHC and SCP. When MCHC was below 330–335 g/L, the risk increased rapidly as MCHC rose, suggesting that this range represents a sensitive clinical threshold for identifying abnormalities in red blood cell quality and for monitoring microcirculatory status in affected children. For children with MCHC near this range, clinicians should strengthen fluid management and consider adjunctive medications to improve microcirculation. At the same time, the possibility of nutritional anemia should be assessed, and potential deficiencies in iron, folic acid, etc., should be corrected. Cholesterol is a core structural component of neuronal cell membranes and myelin within the central nervous system; decreased cholesterol levels may indicate impaired cerebral lipid metabolism and insufficient nutritional reserves. A hypocholesterolemic state may adversely affect neuronal repair, myelin regeneration, and membrane stability (19, 20). RCS analysis further indicated a directional turning point for CHO within the 4.0–4.5 mmol/L range: excessively low levels may reflect insufficient substrates for myelin synthesis, whereas higher levels may be related to metabolic dysregulation. Clinically, attention should focus on whether cholesterol remains within an appropriate range, rather than considering changes in only one direction. This finding supports the rationale for dynamic nutritional intervention in children with spastic cerebral palsy. For example, if CHO levels are below 4.0 mmol/L, a diet rich in essential fatty acids (such as Omega-3) or specific lipid supplements should be considered (21). to facilitate structural and functional recovery of the nervous system. Direct bilirubin is an important endogenous antioxidant. Reduced levels may reflect diminished antioxidant defense capacity and/or increased consumption (22, 23), suggesting a role of oxidative stress in the pathophysiology of SCP (24). When DBIL remains continuously below the lower limit of normal, it is recommended to supplement with antioxidants such as vitamin E and vitamin C, while also monitoring liver function. Taken together, these findings suggest that future management strategies for SCP should move beyond a rehabilitation-only paradigm centered on motor function and toward a multidimensional, individualized precision-intervention framework. Such a framework should integrate targeted nutritional support, appropriately dosed antioxidant approaches, and systematic anti-inflammatory management, while closely monitoring potential adverse effects of medications used for comorbidities.

The nomogram developed in this study is based on seven routine blood test indicators, with an independent test set AUC of 0.972, demonstrating strong discriminative power. Clinicians can input the measured values into the nomogram during each follow-up to calculate the inflammation-metabolism composite load score. By dynamically tracking changes in the score, signals of disease aggravation can be identified, and accordingly, nutritional support, anti-inflammatory treatment, or medication plans can be adjusted. This provides a quantitative reference for the long-term individualized management of SCP children.

With respect to severity-related biomarkers, this study observed an increasing trend in ALT and WBC among children with severe cerebral palsy, whereas ALP showed a weak inverse association, collectively suggesting a high multi-system burden involving the “liver–bone–immune/inflammatory” axis. Elevated ALT typically indicates hepatocellular injury or hepatic dysfunction, which may be attributable to malnutrition, long-term use of antiseizure medications, or other metabolic disturbances commonly observed in children with cerebral palsy; therefore, the possibility of drug-induced liver injury warrants careful attention (25, 26). The weak negative association between ALP and severe cerebral palsy may be explained by prolonged lack of weight-bearing and voluntary physical activity in children with severe impairment (GMFCS levels IV–V). Reduced mechanical loading markedly diminishes osteoblast activity and lowers bone turnover, thereby decreasing the secretion of bone-derived ALP. Children with severe cerebral palsy frequently exhibit osteoporosis and low bone mineral density (27, 28) and often present with malnutrition and deficiencies in trace elements such as zinc. Because ALP is a zinc-dependent enzyme, its activity may be directly influenced by nutritional status. Elevated WBC, in turn, represents a direct manifestation of an intensified systemic inflammatory response. Based on these associations, comprehensive management of children with severe cerebral palsy should also prioritize hepatoprotection, rational pharmacotherapy to minimize hepatic burden, and optimization of nutritional regimens. In addition, intensified rehabilitation training and physical therapy are warranted to reduce fracture risk, alongside proactive supplementation with calcium, vitamin D, and trace elements (29) to support skeletal development and health, as well as more assertive control of systemic inflammation. The subgroup model has an AUC of 0.717, indicating moderate discriminative ability. However, the selected markers—ALT, WBC, and ALP—each have clear clinical management implications and can directly assist in the systematic monitoring and intervention decision-making for children with severe SCP.

Methodologically, this study implemented a multi-stage analytic workflow to enhance the robustness and interpretability of the findings. First, LASSO regression was used to reduce dimensionality and select candidate predictors from the initial feature set. Subsequently, univariable and multivariable logistic regression analyses identified seven potential biomarkers associated with spastic cerebral palsy. RCS were then applied to assess potential nonlinear relationships between key indicators and outcome risk, and an SHAP-based interpretability framework was used to quantify feature contributions. Finally, a nomogram was constructed, and model performance was evaluated using ROC analysis, calibration curves, and DCA, with additional sensitivity analyses conducted using three alternative modeling approaches.

Nevertheless, several limitations of this study should be considered when interpreting the conclusions. Internal validation was performed using an outcome-stratified 7:3 train/test split and 400 bootstrap resamples, and the results indicated good internal stability of the model. The three-model comparisons (GLM, RF, and SVM) presented in the Supplementary Materials further supported this finding. However, it should be noted that all participants were recruited from only two medical institutions in China, and external validation in independent multicenter cohorts is lacking. Therefore, the reported model performance may be overestimated relative to its true generalizability in broader populations. Future studies should validate the model’s generalization performance and clinical applicability in multicenter, prospective external cohorts. In addition, this was a retrospective study, and the original medical records did not contain detailed information on medication use (e.g., types, dosages, and treatment duration of antiseizure drugs and muscle relaxants) or standardized assessments of nutritional status (e.g., dietary intake records, micronutrient testing, and grading systems for feeding and drinking ability). Consequently, we were unable to further stratify analyses to evaluate the effects of medication exposure and nutritional status on hematological parameters in children with cerebral palsy. We recommend that future prospective studies systematically collect medication regimens and standardized nutritional assessment data to clarify the independent contributions of these factors to hematological indices, thereby providing evidence to support more precise intervention strategies.

## 5 Conclusion

In summary, by integrating routinely available clinical parameters and applying interpretable machine-learning approaches, this study systematically delineated a distinct inflammation- and metabolism-related biomarker profile that differentiates children with SCP from healthy controls. Among these biomarkers, MPV demonstrated the highest predictive value for discriminating disease status. In addition, ALT, ALP, and WBC count were identified as independent biomarkers associated with the severity of motor functional impairment. Collectively, these findings provide new empirical evidence for understanding the systemic pathophysiological dysregulation in SCP and establish an important theoretical basis for developing adjunctive diagnostic tools, implementing disease monitoring, and formulating multidimensional, individualized treatment strategies.

However, the conclusions of this study are constrained by its cross-sectional design, recruitment from a limited population source, and potential unmeasured confounding. Therefore, the observed biomarker associations require validation in larger, multicenter, prospective cohort studies and across broader population settings. Future research should aim to deepen mechanistic understanding of these biomarkers and to investigate their longitudinal relationships with clinical outcomes, ultimately advancing SCP management toward more precise and systematic care.

## Data Availability

The original contributions presented in the study are included in the article/Supplementary material, further inquiries can be directed to the corresponding author/s.
