# Supplementary material for: Machine learning—based analysis of blood biomarker features in spastic cerebral palsy and their clinical significance
Source: Front Neurol. 2026 Mar 23;17:1797944. doi: 10.3389/fneur.2026.1797944 (PMC13050735; doi:10.3389/fneur.2026.1797944)

Table S1. baseline characteristics

Variable	Overall (n=488)	CONTROL (n=158)	SCP (n=330)	P-value	SMD
Male, n (%)	323 (66.19%)	92 (58.23%)	231 (70.00%)	0.010	0.247
Age	7.03 ± 2.57	6.74 ± 2.37	7.16 ± 2.65	0.077	0.168
WBC	7.43 ± 2.22	6.86 ± 1.79	7.71 ± 2.36	<0.001	0.402
NEUT (×10^9/L)	3.75 ± 1.92	3.23 ± 1.52	4.00 ± 2.04	<0.001	0.423
LYMPH (×10^9/L)	3.12 ± 2.80	2.93 ± 0.92	3.22 ± 3.34	0.150	0.116
MONO (×10^9/L)	0.50 ± 0.31	0.46 ± 0.18	0.51 ± 0.35	0.057	0.164
EOS (×10^9/L)	0.19 ± 0.19	0.21 ± 0.15	0.19 ± 0.21	0.308	0.093
BASO (×10^9/L)	0.04 ± 0.07	0.03 ± 0.02	0.05 ± 0.09	<0.001	0.274
NEUT%	48.94 ± 13.30	45.93 ± 11.97	50.38 ± 13.68	<0.001	0.347
LYMPH%	41.16 ± 12.48	43.76 ± 11.29	39.92 ± 12.85	<0.001	0.318
MONO%	6.65 ± 1.99	6.83 ± 2.18	6.57 ± 1.89	0.191	0.130
EOS%	2.64 ± 2.39	3.04 ± 2.09	2.45 ± 2.49	0.006	0.259
BASO%	0.62 ± 0.92	0.49 ± 0.58	0.67 ± 1.04	0.013	0.218
RBC	4.64 ± 0.38	4.54 ± 0.33	4.68 ± 0.40	<0.001	0.369
HGB	130.41 ± 10.59	127.85 ± 8.95	131.63 ± 11.10	<0.001	0.375
HCT	38.37 ± 5.25	38.63 ± 2.66	38.25 ± 6.14	0.333	0.082
MCV	83.85 ± 4.07	84.99 ± 3.83	83.31 ± 4.07	<0.001	0.425
MCH	28.67 ± 11.09	29.70 ± 19.36	28.17 ± 1.55	0.325	0.111
MCHC	335.84 ± 8.07	330.97 ± 7.78	338.17 ± 7.12	<0.001	0.966
RDW	13.24 ± 1.15	13.33 ± 1.27	13.19 ± 1.09	0.238	0.117
PLT	288.49 ± 74.42	284.34 ± 69.46	290.47 ± 76.71	0.379	0.084
MPV	8.95 ± 1.45	10.19 ± 1.34	8.36 ± 1.08	<0.001	1.507
PDW	15.56 ± 1.65	14.29 ± 2.37	16.17 ± 0.48	<0.001	1.097
TBIL	9.41 ± 6.68	8.79 ± 4.19	9.71 ± 7.57	0.086	0.150
DBIL	2.08 ± 1.20	2.72 ± 1.32	1.51 ± 0.72	<0.001	1.135
IBIL	7.05 ± 6.30	6.90 ± 8.30	7.18 ± 3.72	0.698	0.043
TP	71.67 ± 29.02	70.93 ± 50.43	72.03 ± 5.74	0.786	0.030
ALB	44.46 ± 3.32	44.16 ± 2.85	44.61 ± 3.52	0.136	0.139
GLB	25.93 ± 4.32	22.83 ± 3.51	27.42 ± 3.85	<0.001	1.246
ALT	18.21 ± 12.74	15.65 ± 8.52	19.44 ± 14.19	<0.001	0.324
AST	32.31 ± 94.54	28.39 ± 8.96	34.20 ± 114.86	0.362	0.071
ALP	241.17 ± 71.44	267.72 ± 82.47	228.47 ± 61.72	<0.001	0.539
GGT	13.90 ± 10.85	13.96 ± 12.53	13.88 ± 9.96	0.942	0.007
CHO	3.93 ± 0.67	4.06 ± 0.64	3.79 ± 0.67	<0.001	0.413
Urea	4.63 ± 1.17	4.50 ± 1.08	4.69 ± 1.20	0.077	0.169
Cr	38.46 ± 8.85	38.48 ± 7.98	38.45 ± 9.25	0.976	0.003
NLR	1.53 ± 1.44	1.29 ± 1.26	1.65 ± 1.51	0.007	0.254
PLR	108.80 ± 48.11	106.15 ± 43.73	110.07 ± 50.09	0.378	0.083
SII	440.75 ± 440.98	371.33 ± 375.07	473.98 ± 466.16	0.009	0.243
SIRI	0.79 ± 0.92	0.63 ± 0.71	0.86 ± 1.00	0.003	0.268

RPR	0.05 ± 0.02	0.05 ± 0.02	0.05 ± 0.02	0.338	0.095
MPR	0.03 ± 0.02	0.04 ± 0.02	0.03 ± 0.01	<0.001	0.478
NMR	8.12 ± 4.10	7.34 ± 3.27	8.49 ± 4.39	0.001	0.298
NAR	0.08 ± 0.04	0.07 ± 0.03	0.09 ± 0.05	<0.001	0.417
MLR	0.18 ± 0.10	0.17 ± 0.09	0.19 ± 0.11	0.101	0.154
AISI	227.53 ± 276.08	183.89 ± 224.59	248.43 ± 295.64	0.008	0.246

Figure S1. LASSO Coefficient Profiles Using the λ.min Penalty Parameter

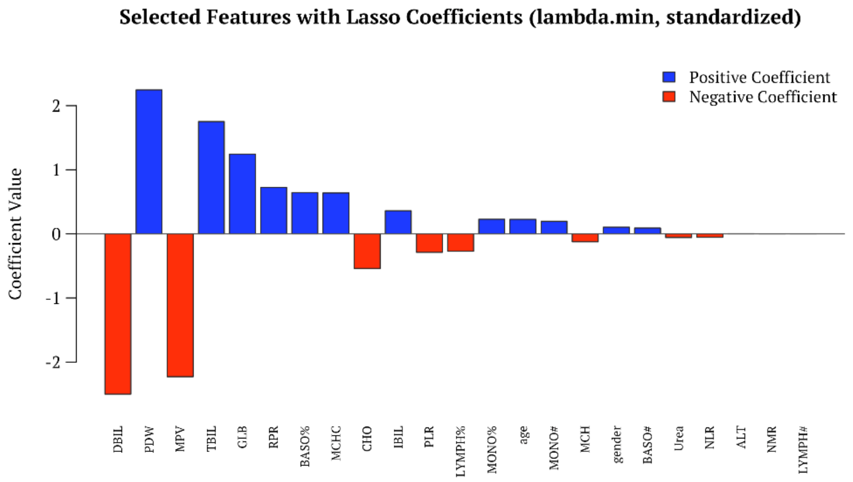

Figure S2. Results of Univariable Logistic Regression Analyses

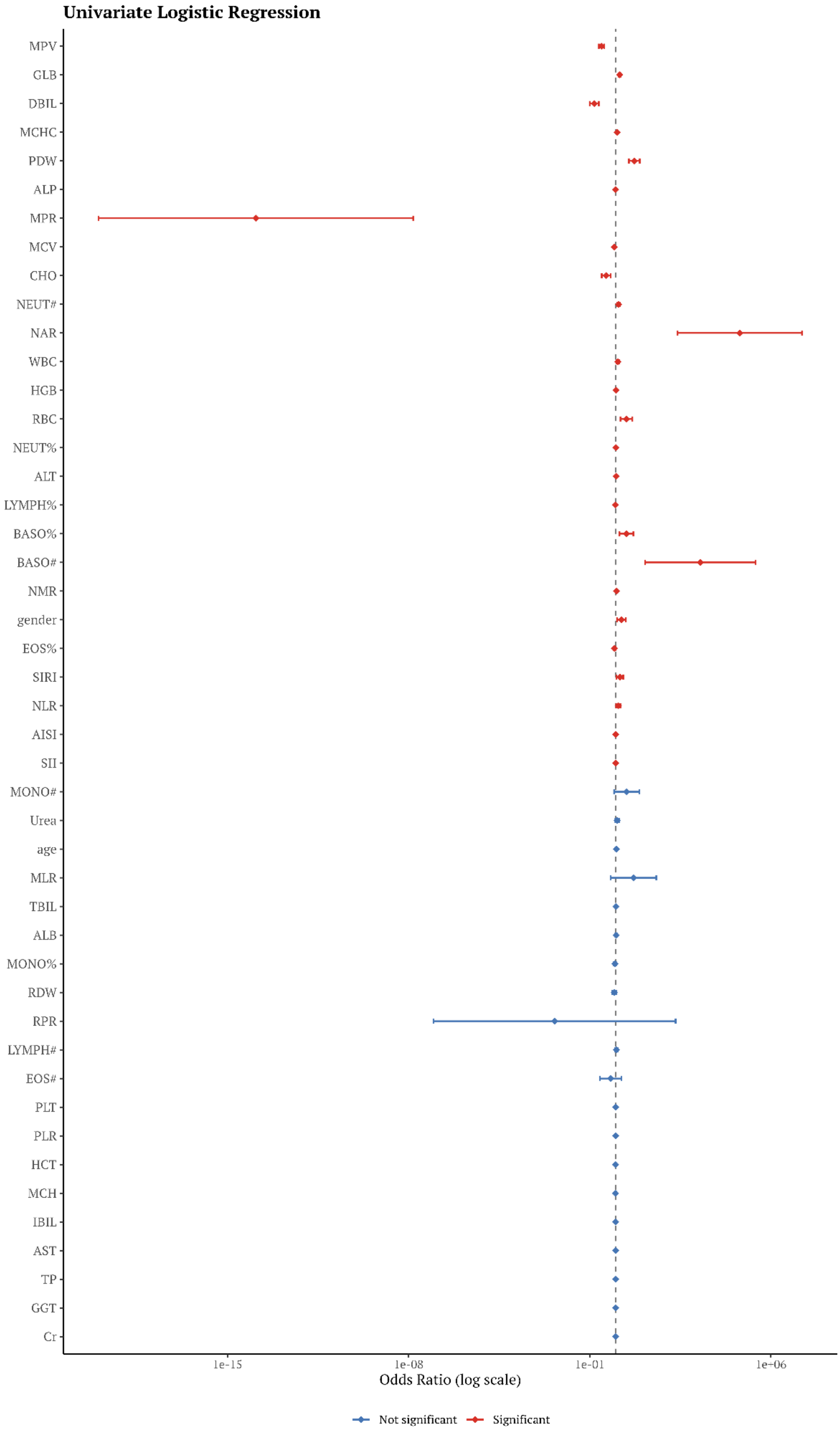

Figure S3. Results of Multivariable Logistic Regression Analyses

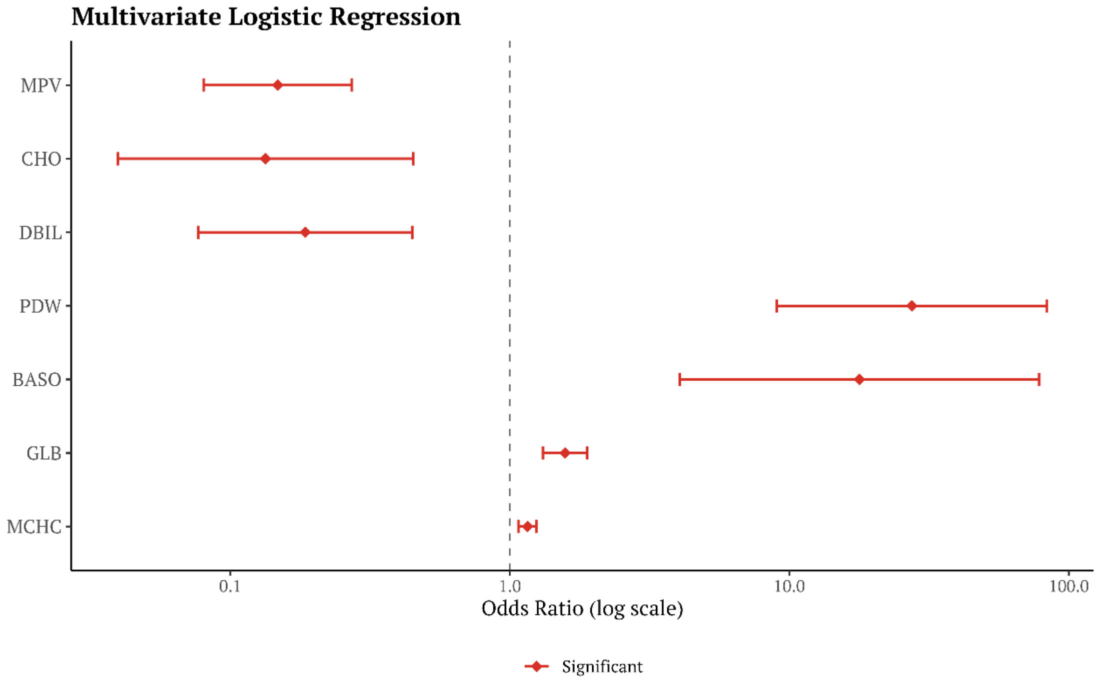

Figure S4. SHAP feature importance（Bar）

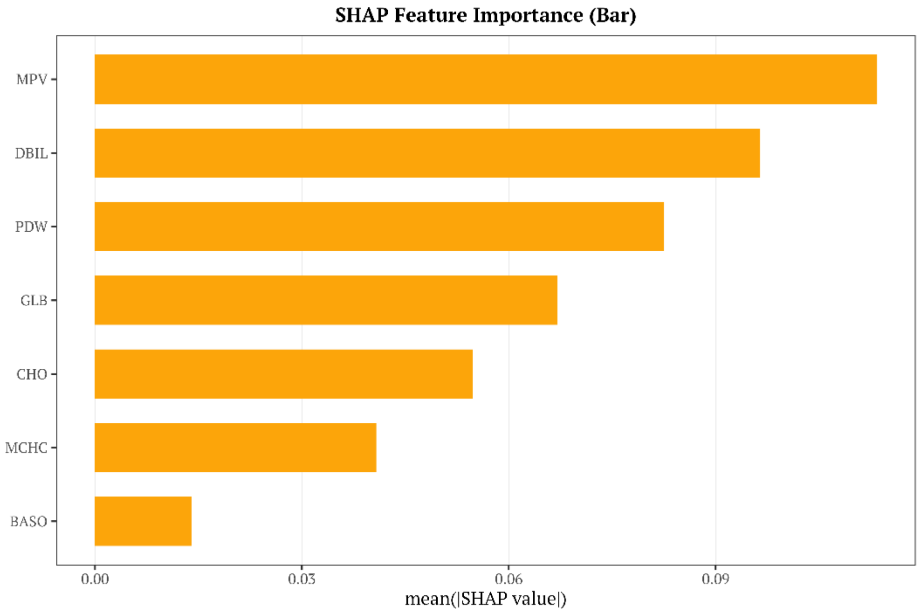

Figure S5. SHAP beeswarm

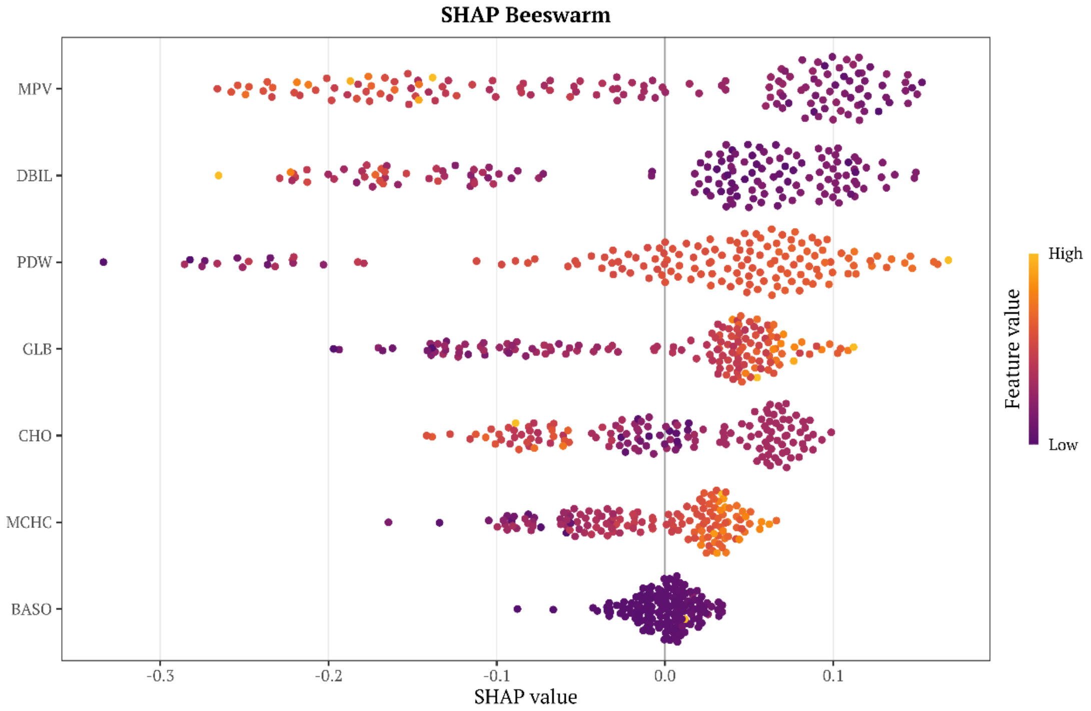

Figure S6. SHAP dependence

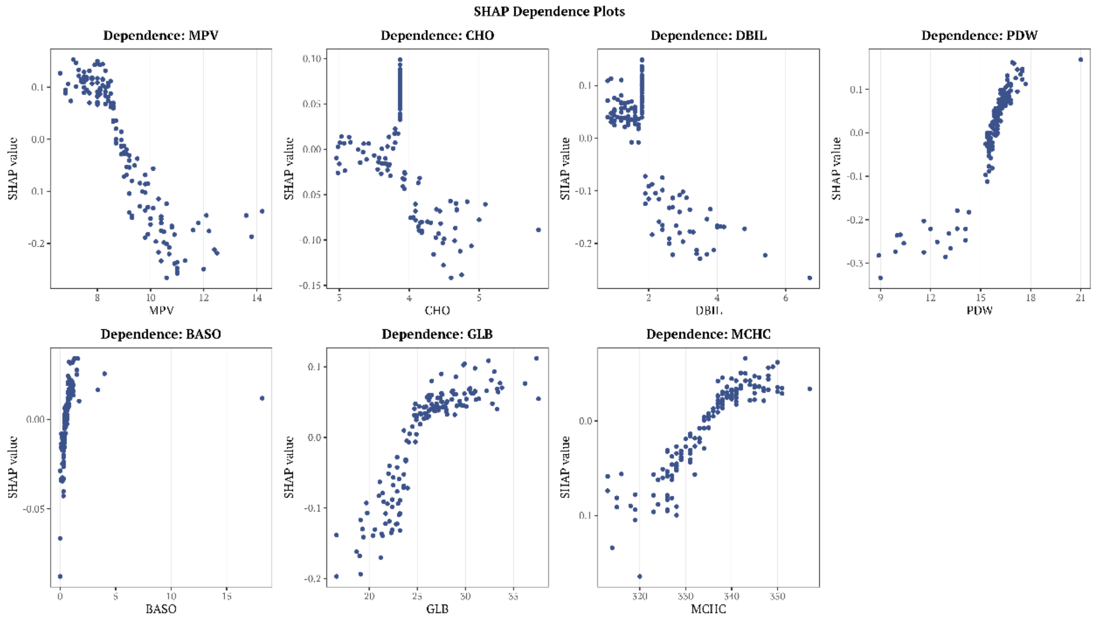

Figure S7. Decision Curve Analysis (DCA) for the Primary Model

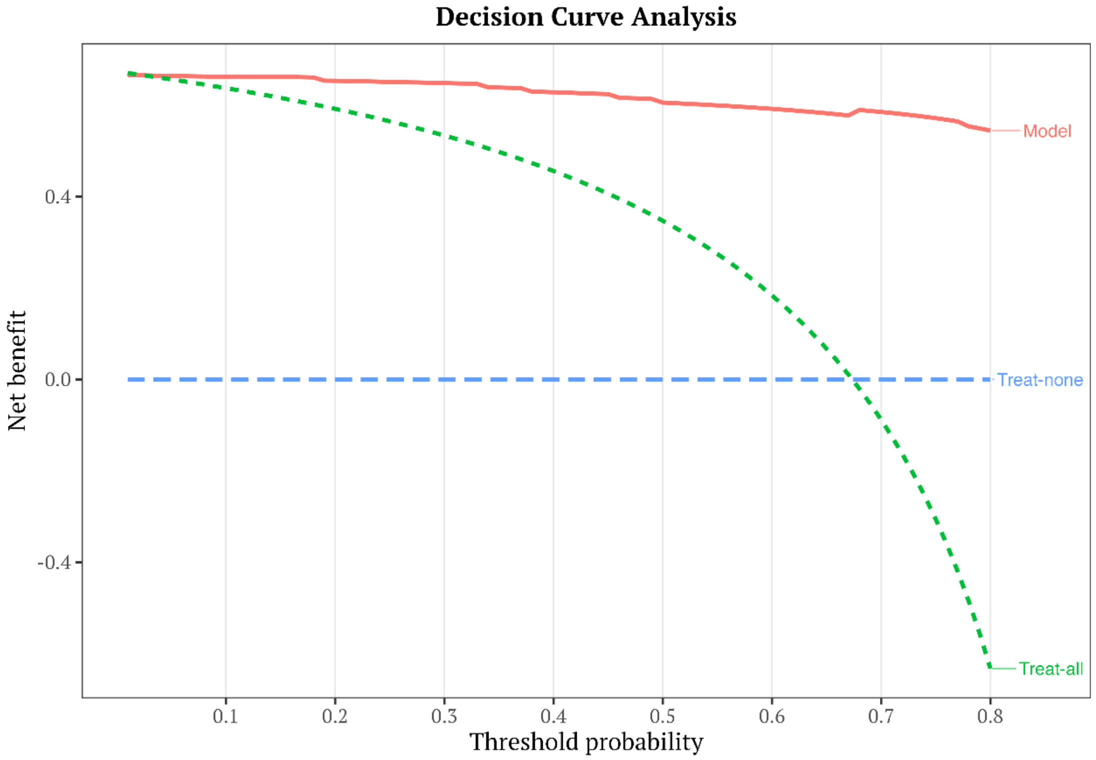

Figure S8. Main model predicted probability distribution

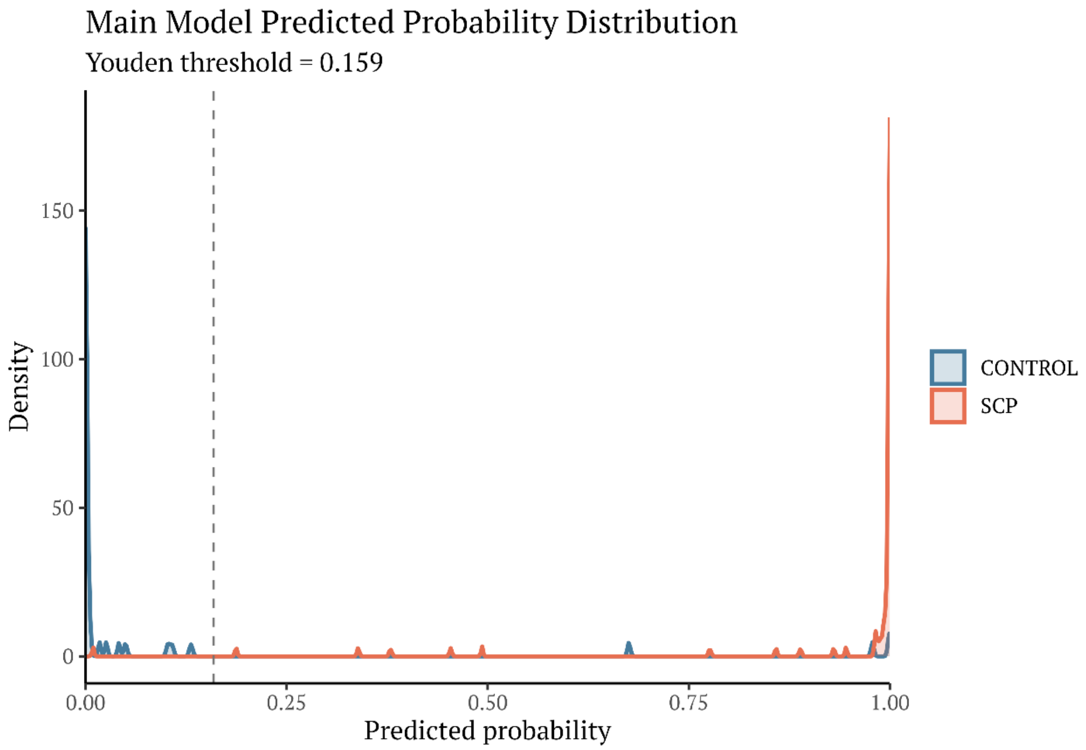

Figure S9. Main model confusion matrix

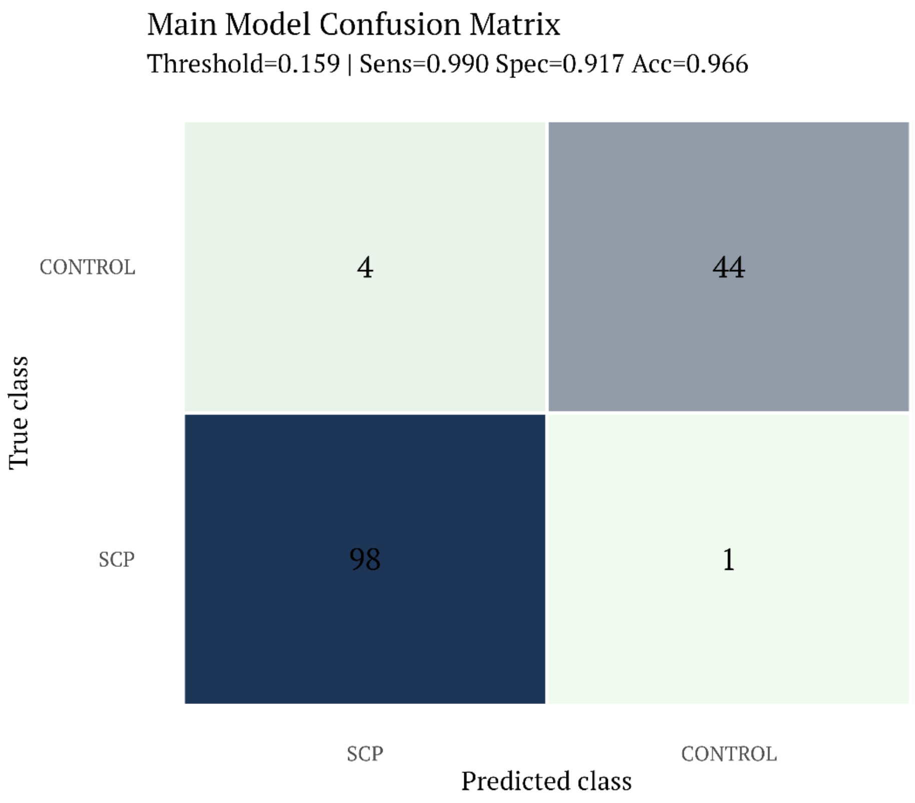

Figure S10. ROC Curve of the Subgroup Model (Severe vs. Mild) in the Independent Test Set (n = 99)

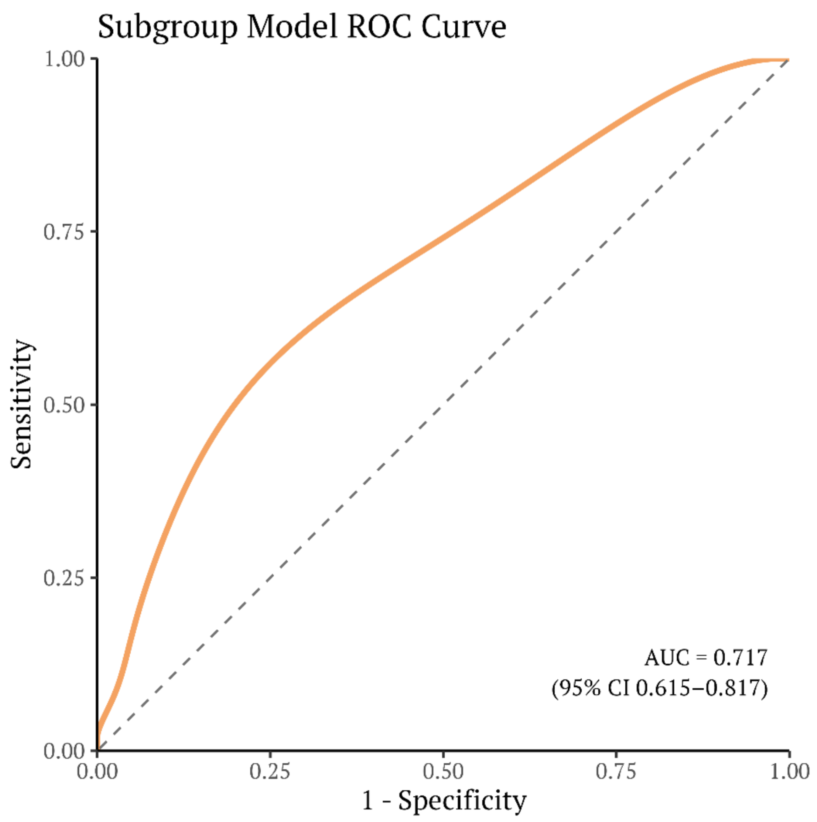

Figure S11. Distribution of Predicted Probabilities of the Subgroup Model in the Test Set

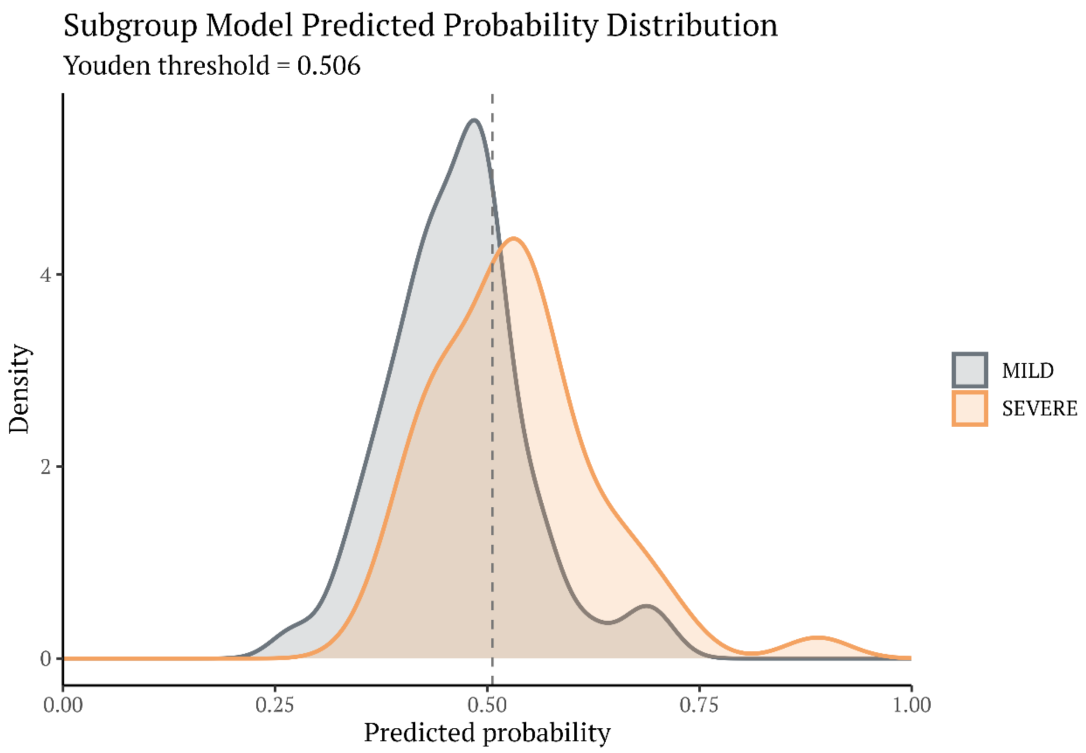

Figure S12. Confusion Matrix of the Subgroup Model in the Test Set

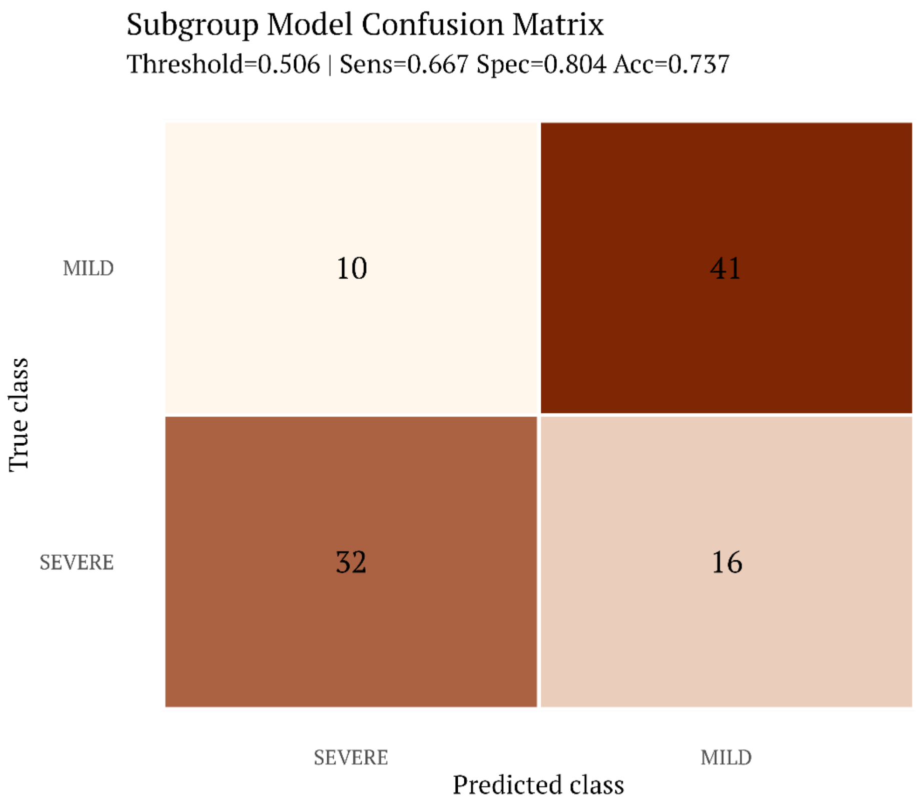

Figure S13. Calibration Curve of the Subgroup Model in the Test Set

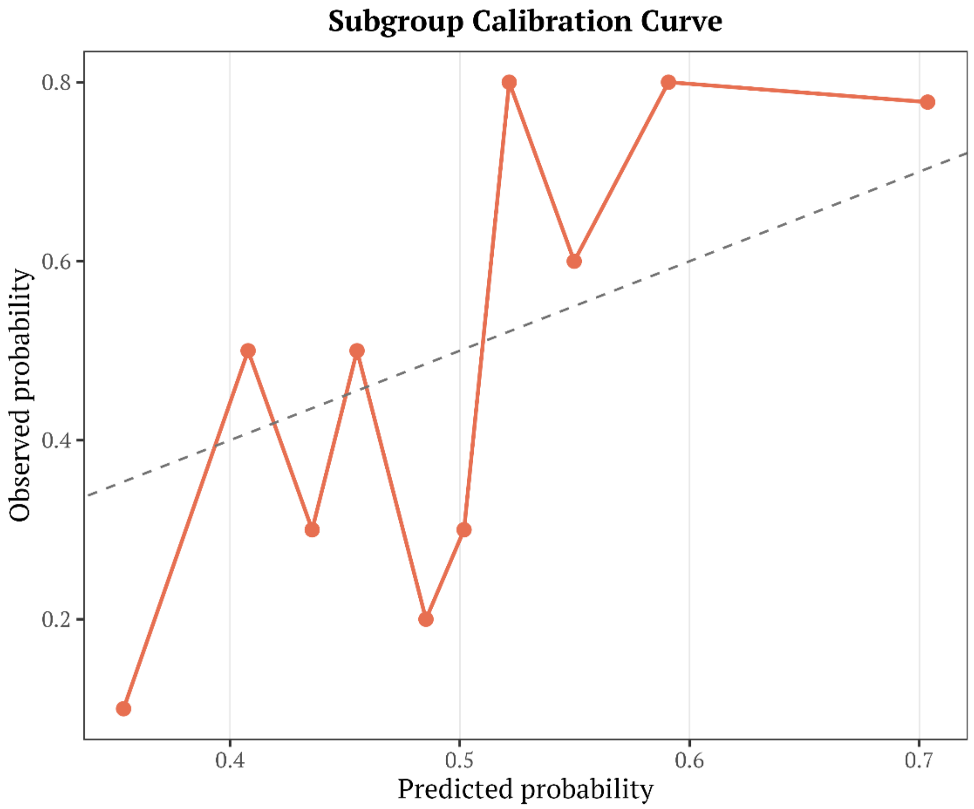

Figure S14. Nomogram of the Subgroup Model

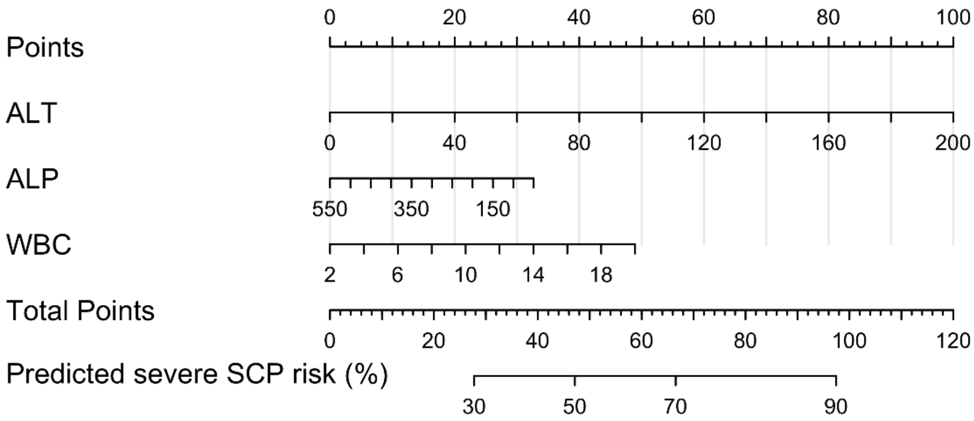

Figure S15. Variable Contributions and Risk Mapping in the Subgroup Nomogram

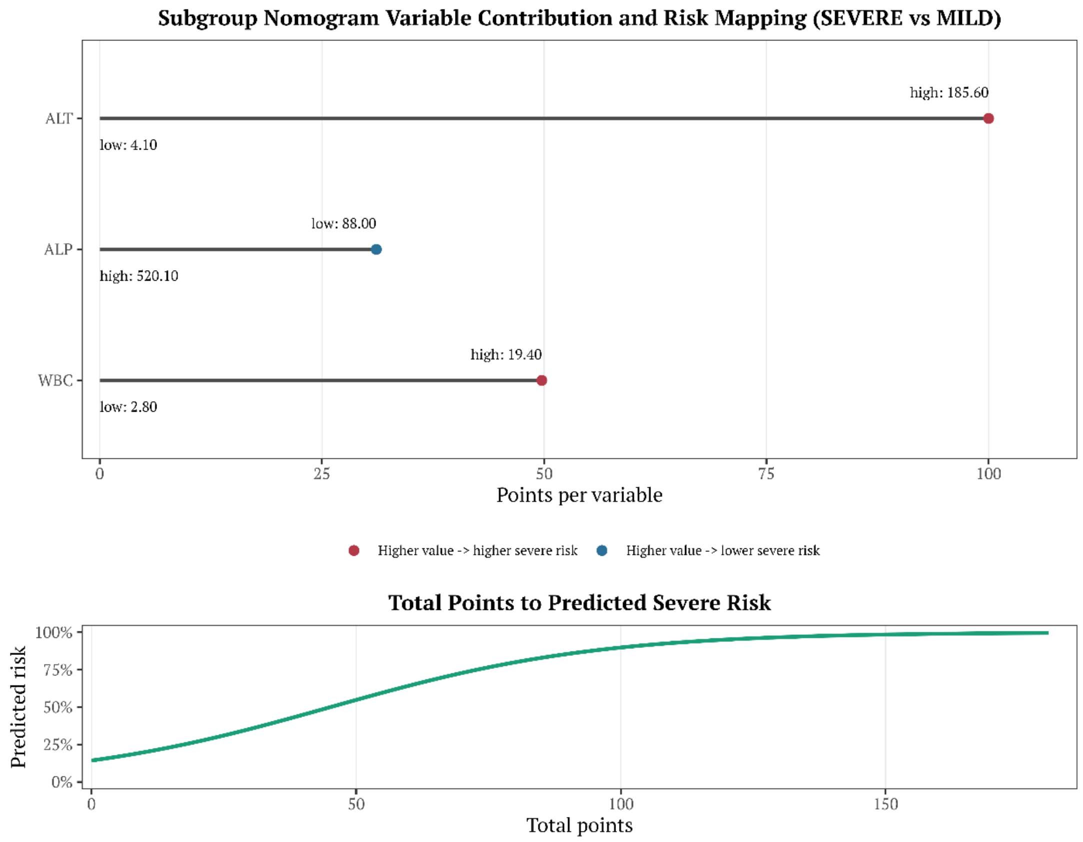

Figure S16. Decision Curve Analysis (DCA) for the Subgroup Model

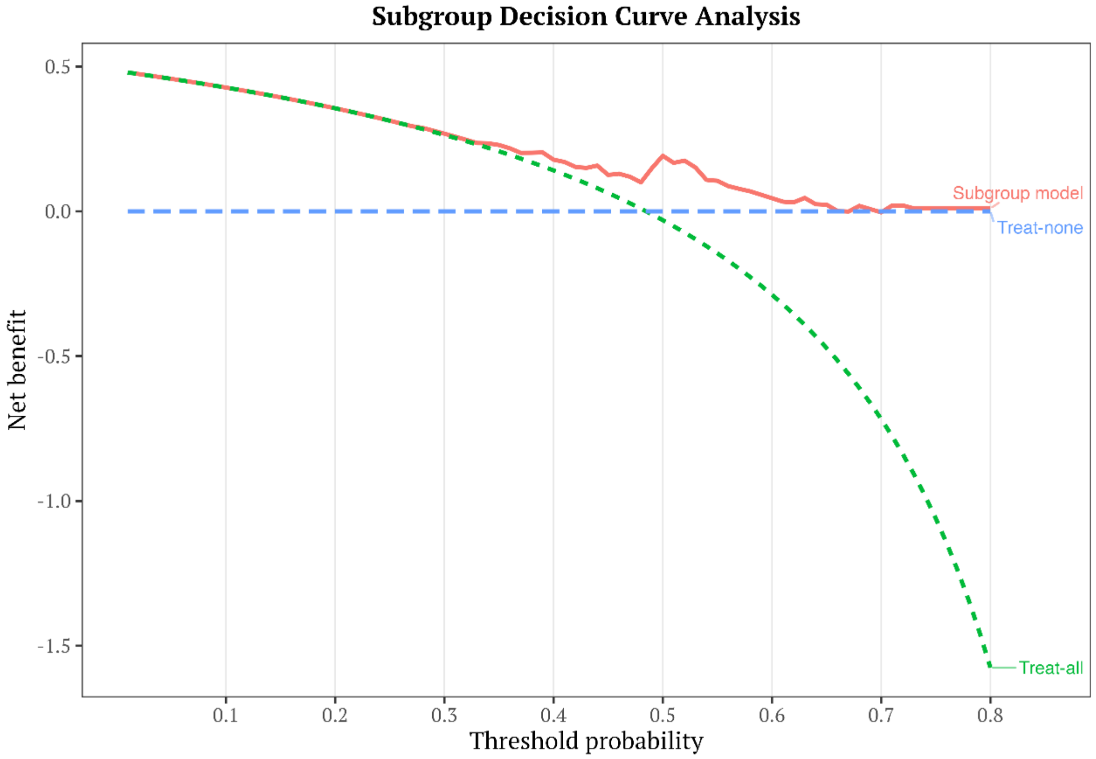

Figure S17. Comparison of ROC Curves for GLM, RF, and SVM in the Test Set of the Primary Model

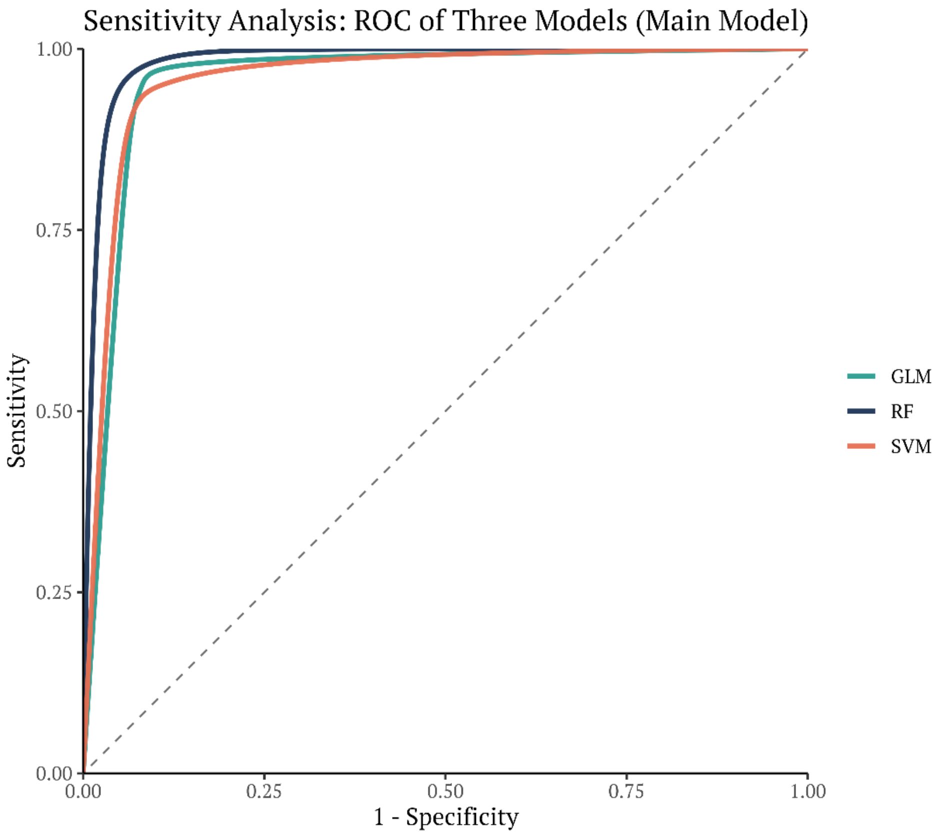

Figure S18. Comparison of AUCs and 95% Confidence Intervals for the Three Models in the Test Set of the Primary Model

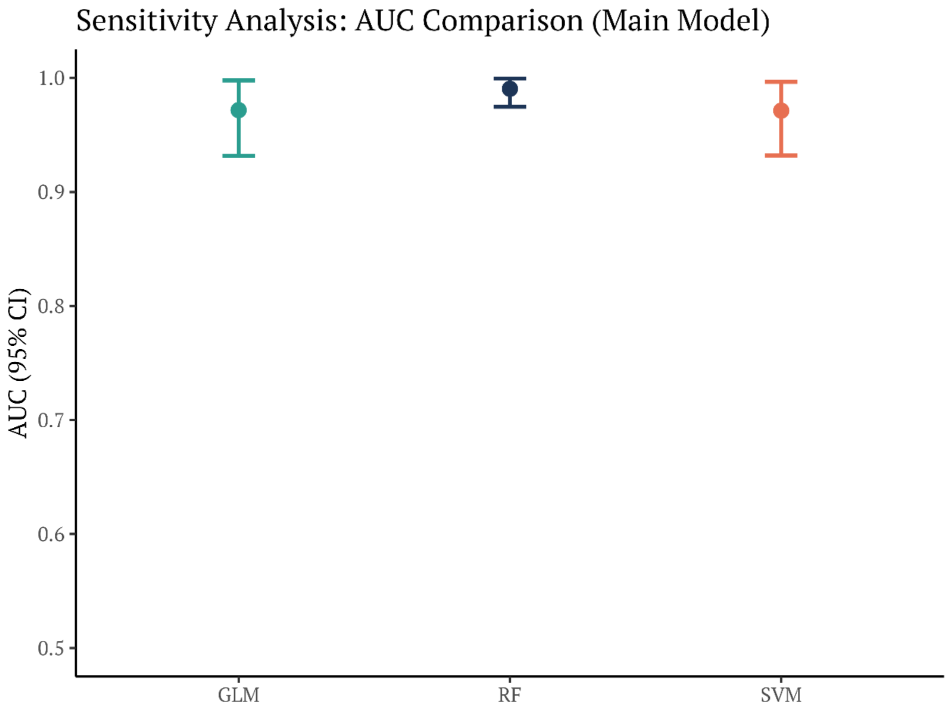

Figure S19. Comparison of ROC Curves for GLM, RF, and SVM in the Test Set of the Subgroup Model

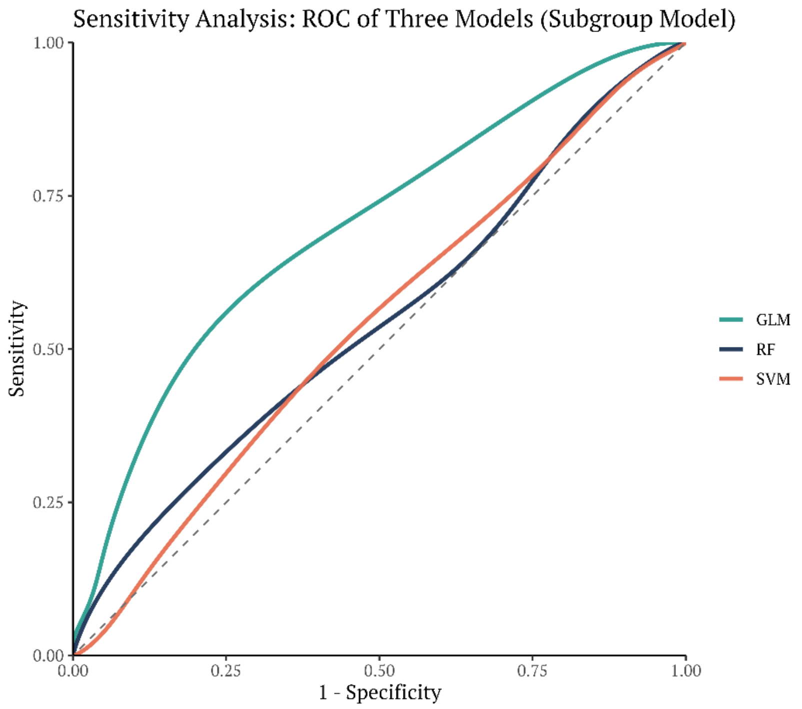

Figure S20. Comparison of AUCs and 95% Confidence Intervals for the Three Models in the Test Set of the Subgroup Model

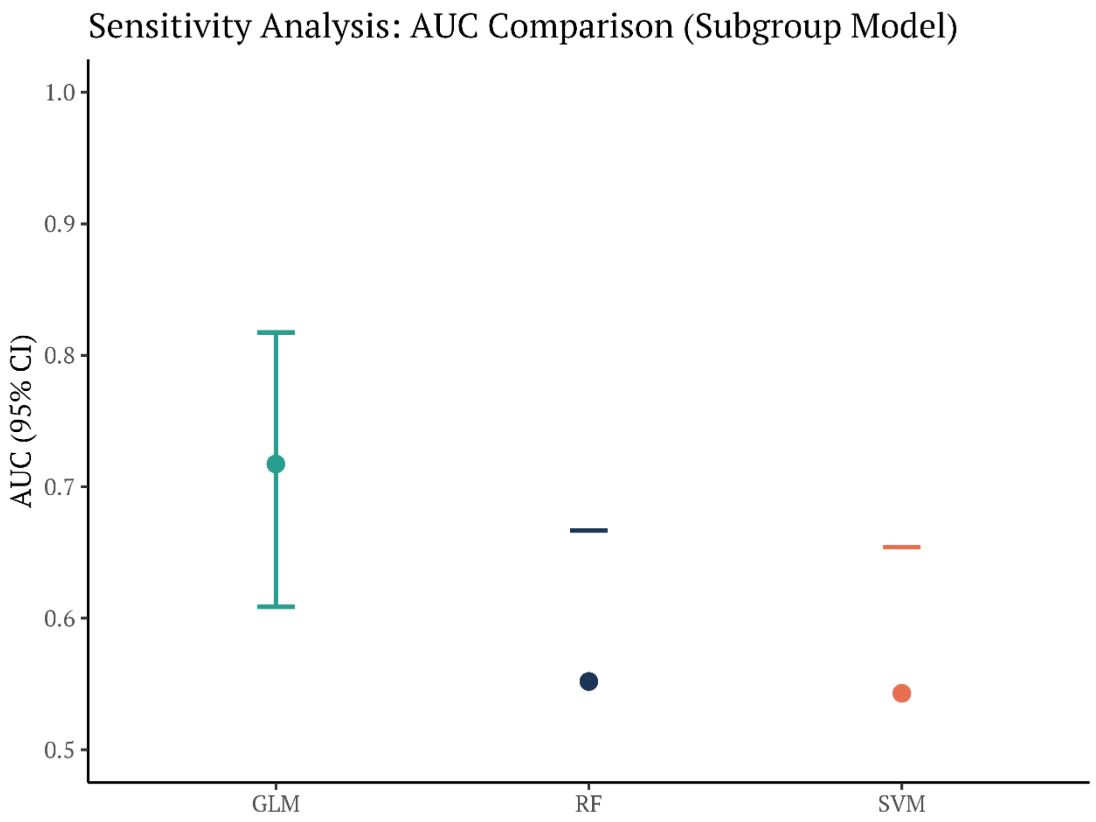

Supplement: Supplementary file 1 [file Data_Sheet_1.pdf]
